# Epithelial-to-mesenchymal transition, inflammation, subsequent collagen production, and reduced proteinase expression cooperatively contribute to cyclosporin-A-induced gingival overgrowth development

**DOI:** 10.3389/fphys.2023.1298813

**Published:** 2023-12-13

**Authors:** Mio Imagawa, Takanori Shinjo, Kohei Sato, Kentaro Kawakami, Tatsuro Zeze, Yuki Nishimura, Masaaki Toyoda, Shuang Chen, Naoaki Ryo, Al-kafee Ahmed, Misaki Iwashita, Akiko Yamashita, Takao Fukuda, Terukazu Sanui, Fusanori Nishimura

**Affiliations:** ^1^ Section of Periodontology, Faculty of Dental Science, Kyushu University, Fukuoka, Japan; ^2^ Department of Periodontology and Endodontology, Nagasaki University Graduate School of Biomedical Sciences, Nagasaki, Japan

**Keywords:** SPOCK-1, drug-induced gingival overgrowth, periodontitis, cyclosporin-A, epithelial-tomesenchymal transition

## Abstract

Drug-induced gingival overgrowth (DIGO), induced by certain immunosuppressive drugs, antihypertensive agents, and antiepileptic drugs, may contribute to the formation of deeper periodontal pockets and intractableness in periodontitis. To date, multiple factors such as enhanced matrix production, inflammation, and reduced matrix degradation might be involved in the pathogenesis of DIGO. We have previously reported that SPOCK-1, a heparan sulfate proteoglycan, could affect gingival thickening by promoting epithelial-to-mesenchymal transition (EMT) in gingival keratinocytes. However, few studies have investigated whether a combination of these factors enhances the DIGO phenotype in animal models. Therefore, we investigated whether SPOCK-1, periodontal inflammation, and cyclosporin-A (CsA) could cooperatively promote gingival overgrowth. We first confirmed that Spock-1 overexpressing (Spock1-Tg) mice showed significantly thicker gingiva and greater alveolar bone loss than WT mice in response to ligature-induced experimental periodontitis. DIGO was induced by the combination of CsA administration and experimental periodontitis was significantly enhanced in Spock1-Tg mice compared to that in WT mice. Ligature-induced alveolar bone loss in CsA-treated Spock1-Tg mice was also significantly greater than that in CsA-treated WT mice, while being accompanied by an increase in *Rankl* and *Col1a1* levels and a reduction in matrix metalloprotease expression. Lastly, SPOCK-1 promoted RANKL-induced osteoclast differentiation in both human peripheral blood mononuclear cells and murine macrophages, while peritoneal macrophages from Spock1-Tg mice showed less TNFα and IL-1β secretion than WT mice in response to *Escherichia coli* lipopolysaccharide. These results suggest that EMT, periodontal inflammation, and subsequent enhanced collagen production and reduced proteinase production contribute to CsA-induced DIGO pathogenesis.

## Introduction

Drug-induced gingival overgrowth (DIGO) is caused by certain medications, such as the immunosuppressive drug cyclosporin-A (CsA), antihypertensive Calcium-channel blockers, and the antiepileptic drug phenytoin (Subramani et al., 2013; Sharma et al., 2020; Fang and Tan, 2021; Chojnacka-Purpurowicz et al., 2022). Although several mechanisms underlying the pathogenesis of these side effects have been proposed, many previous studies have focused on single factors, such as enhanced matrix production, the involvement of inflammation, and reduced matrix degradation; however, they have not explored the combination and cooperation of all these events at the molecular level (Nishimura et al., 2002; Kuo et al., 2012; Subramani et al., 2015; Drozdzik A and Drozdzik M, 2023).

We previously reported that transforming growth factor-β (TGF-β)-induced SPOCK-1, one of the heparan sulfate proteoglycans, in the gingival keratinocytes played key roles in inducing DIGO via epithelial to mesenchymal transition (EMT), while showing that SPOCK-1 transgenic (*Spock1*-Tg) mice developed DIGO-like phenotype without any other obvious systemic disorders (Alshargabi et al., 2020). However, the degree of gingival thickening in *Spock1*-Tg mice was very limited; in the case of human DIGO, the enlarged gingival mass of diseased patients is usually quite large and may cover the entire tooth crown (Liu et al., 2022).

Based on these observations, we hypothesized that in addition to EMT, other factors also contribute to the pathogenesis of human DIGO in a cooperative manner. Thus, we aimed to further elucidate the pathogenesis of DIGO by including periodontal inflammation and administering causative drugs to *Spock1*-Tg mice to understand the involvement of these factors in DIGO pathology. Most previous reports have used CsA to implement an experimental DIGO model since CsA is known to induce some degree of DIGO in mice (Hallmon and Rossmann, 1999; Okanobu et al., 2017; Hatano et al., 2021); however, the resulting enlarged gingiva may not cover the entire crown of the tooth. Thus, the reproducibility of this drug in an animal model of DIGO in mice is known. The pharmacological action of CsA is well established. It suppresses calcineurin (CN)/nuclear factor of activated T cell (NF-AT) signaling, thereby suppressing interleukin-2 production in T lymphocytes; this is followed by the suppression of subsequent immune responses (Nishiyama et al., 2004). In addition, previous studies also demonstrated that placing a silk ligature around the molars could successfully induce experimental periodontitis, leading to alveolar bone resorption, similar to human chronic inflammatory periodontal disease (Marchesan et al., 2018). Furthermore, *in vivo* and *in vitro* studies have demonstrated that the combination of CsA with ligature-induced experimental periodontitis effectively elicits DIGO, with the tooth crown being almost completely covered by enlarged gingiva via collagen accumulation due to the downregulation of the CN/NF-AT axis in mice (Okanobu et al., 2017; Hatano et al., 2021).

Therefore, in this study, we combined the DIGO model induced by ligature-induced periodontitis and CsA administration with the EMT model overexpressing SPOCK-1, which was established in our previous study, to evaluate the degree of gingival thickening, alveolar bone resorption, and related gene expression in mice and understand the roles and interactions of periodontal infection/inflammation, the CN/NF-AT axis, and EMT in the pathogenesis of DIGO. Here, we report that all these events cooperatively contribute to disease development.

## Materials and methods

### Reagents

Antibodies against MPO (ab208670) and goat anti-rabbit IgG conjugated to Alexa Fluor 488 (ab150077) were purchased from Abcam (Cambridge, United Kingdom). *E. coli* LPS (O111:B4, L4391) was purchased from Sigma-Aldrich (St. Louis, MO, United States). All the other reagents were of analytical grade. The dilutions used for each application are listed in Supplementary Table S1.

### Mice

Global mouse *Spock1*-overexpressing transgenic (*Spock1*-Tg) mice were generated as described previously (Alshargabi et al., 2020). Briefly, *Spock1* ^*+/−*^ and *Spock1* ^*−/−*^ (wild-type: WT) mice were crossed to produce litters. Tail samples were collected from 4-week-old mice to extract DNA using the KAPA Express Extract (Kapa Biosystems, MA, USA). Genotyping was performed using KOD FX Neo (TOYOBO, Osaka, Japan). Genotyping primer sequences are as follows: pCAGGS-F1:5′-GTCGACATTGATTATTGACTAGTTATTAAT-3′, pCAGGS-R2275:5′-GTCGAGGATCTCCATAAGAAGAGGGACA-3’. We complied with the updated ARRIVE 2.0 guidelines for animal studies. All animal experiments and euthanasia protocols were approved by the Institutional Animal Care and Use Committee of Kyushu University (A23-061-2 and A20-109-2). All mice were euthanized by injecting anesthetic containing 4 mg/kg midazolam (LF6476; SANDOZ, Tokyo, Japan), 5 mg/kg Vetorphale^®^ (Meiji Seika Pharma, Tokyo, Japan), and 0.3 mg/kg Dorbene^®^ (9021; Kyouritsu Seiyaku, Tokyo, Japan) using 1 mL syringe and then blood loss by heart puncture. WT and *Spock1*-Tg mice were cohoused under climate-controlled conditions with a 12-h light/dark cycle and water and food provided *ad libitum*.

### Induction of ligature-induced experimental periodontitis

Male WT and *Spock1*-Tg mice (10-week-old) were randomly divided into those with or without ligature-induced periodontitis. All mice were intraperitoneally injected with anesthetic containing 4 mg/kg midazolam, 5 mg/kg Vetorphale^®^, and 0.3 mg/kg Dorbene^®^ using 1 mL syringe. After confirming loss of consciousness, 6–0 silk thread (Akiyama MEDICAL MEG, Tokyo, Japan) was tied around the left maxillary second M to induce experimental periodontitis. Right maxillae without ligature were used as the un-ligated group. Female WT and *Spock1*-Tg mice were not used in this study to avoid potential sexual cycle impacts. Ten days after the induction of experimental periodontitis, the mice were euthanized, and the maxillae were removed for further analysis. All mice injured during fighting and those with lost ligatures were excluded.

The maxillae were photographed to evaluate gingival thickening. Then, for alveolar bone loss measurements, right (without ligature) and left hemisected maxillae (with ligature) were immersed in 0.5% sodium hypochlorite solution (FUJIFILM, Osaka, Japan) for 3 days, and 3% hydrogen peroxide (FUJIFILM) for 1 day after collecting gingiva around the molars in ISOGENⅡ reagent (NIPPON GENE, Tokyo, Japan) to remove soft tissues. For the histological assays, the right and left hemisected maxillae were fixed in 10% Formalin Neutral Buffer Solution (FUJIFILM) for 24 h and decalcified for 48 h using Osteosoft^®^ (Merck, Darmstadt, Germany). Decalcified maxillae were embedded in paraffin for further analysis.

### Evaluation of gingival thickening

Gingival thickening was evaluated in the occlusal view as previously reported (Okanobu et al., 2017). Briefly, the gingiva around the second M was observed and photographed using a stereomicroscope MZ10F (Leica Microsystems, Wetzlar, Germany). The degree of occlusal gingival overgrowth was calculated from the average distance of the mesiodistal diameter of the second M and the distance from the center of the buccal dental margin to the end of the gingival margin using ImageJ software (National Institutes of Health, Bethesda, MA, USA).

Gingival thickening in the buccal view was assessed based on Okanobu’s method with modifications, as shown in Supplementary Figure S1. Briefly, the buccal gingival overgrowth degree of DIGO was calculated from the average distance between the mesiodistal diameter of the second M and the average distance from the top of the proximal and distal interdental papillae to the mucogingival cheek junction.

### Assessment of experimental periodontitis-induced alveolar bone loss

The defleshed maxillae were washed with PBS and stained with 0.05% toluidine blue (Muto Pure Chemicals, Tokyo, Japan). Photographs were taken using a Leica MZ10 F microscope. Alveolar bone loss was assessed by measuring the cemento-enamel junction (CEJ) of the third mesial root to the pinnacle of the alveolar bone (AB) and the distal and mesial roots of the second and first molars using ImageJ software. Alveolar bone loss in each group was defined as the sum of the distances from the five sites or root areas in the three maxillary molars based on a previous study (Shinjo et al., 2023; Zeze et al., 2023).

### Periodontitis-complicated drug-induced gingival overgrowth model

Male WT and *Spock1*-Tg mice (9–10-week-old) were subjected to gingival hyperplasia with a combination of cyclosporin-A (CsA; Tokyo Chemical Industry, Tokyo, Japan) and ligature-induced periodontitis according to a previous report (Okanobu et al., 2017) with modifications. Briefly, WT and *Spock1*-Tg mice were intraperitoneally administered 50 mg/kg CsA or the same amount of polyoxyethylene Castor Oil (FUJIFILM) mixed with 99.5% ethanol (FUJIFILM) for 7 consecutive days. The mice were then divided into those with or without ligature-induced experimental periodontitis using 6–0 silk ligatures at the maxillary second M under same anesthesia as described in the section of ‘Induction of ligature-induced experimental periodontitis’. CsA or vehicle was administered intraperitoneally for 14 days after the induction of experimental periodontitis to create a DIGO model of periodontitis. Finally, male WT and *Spock1*-Tg mice were divided into eight groups: (1) WT vehicle without ligature (lig-), (2) *Spock1*-Tg vehicle lig-, (3) WT vehicle with ligature (lig+), (4) *Spock1*-Tg vehicle lig+, (5) WT CsA lig-, (6) *Spock1*-Tg CsA lig-, and (7) WT CsA lig+, (8) *Spock1*-Tg CsA lig+. The euthanized mice were photographed to evaluate gingival overgrowth and the maxillae were hemisected for further analysis, as described above.

### RNA extraction and quantitative real-time PCR

The gingiva of the right (lig-) and left (lig+) maxillary tissues were harvested and promptly immersed in ISOGEN II for total RNA extraction. cDNA was synthesized using the PrimeScript RT Master Mix (Takara Bio, Otsu, Japan). Quantitative reverse transcriptional polymerase chain reaction (qRT-PCR) was performed using Luna Universal qPCR Master Mix (New England BioLabs, Ipswich, MA, USA) on a StepOne Plus™ Real-Time System (Applied Biosystems, Carlsbad, CA, USA) under the following conditions: 95 °C for 3 min, 40 cycles of 95 °C for 3 s, and 60 °C for 30 s. A passive reference dye (Rox) was added to the PCR master mix to correct for varying copies of first-strand cDNA templates. The results were recorded and analyzed using StepOne™ software V2.2.2 (Applied Biosystems) utilizing an auto-calculated threshold cycle. The ΔΔCT method was used to calculate the relative expression levels of the individual genes, and 18s rRNA was used as an internal control. Primer sequences used in this study are listed in Supplementary Table S2.

### Histological section

The decalcified maxillae were embedded in paraffin and sectioned at a thickness of 5 µm to prepare tissue sections. Masson’s Trichrome staining was performed as described previously (Alshargabi et al., 2020).

### Western blotting analysis

The gingiva of the right (lig-) and left (lig+) maxillary tissues from WT and *Spock1*-Tg mice with or without CsA administration were harvested, snap frozen with dry ice, ground with a hammer, then homogenized in RIPA buffer (Nacalai Tesque, Kyoto, Japan) supplemented with protease inhibitor mixture (1:100 dilution; 25955-11, Nacalai Tesque) and phosphatase inhibitor mixture (1:100 dilution; 78428, Sigma-Aldrich). Protein concentrations were determined using the BCA Protein assay kit (Thermo Fisher Scientific, Waltham, MA USA). Lysates with a fixed protein concentration were subjected to SDS/PAGE, then gels were transferred to PVDF membranes. After blocking with 5% skim milk, membranes were incubated with primary antibodies at 4 °C overnight and with appropriate secondary antibodies for 1 h. Membranes were developed using a chemiluminescent Chemi-Lumi One Super (02230-30; Nacalai Tesque). Blots were scanned using Image Quant LAS800 (GE healthcare, Chicago, IL, USA) and analyzed using ImageJ software (National Institute of Health, Bethesda, MA, USA). Antibody concentrations for Western blotting (WB) are listed in Supplementary Table S1.

### TRAP staining

Each tissue section was stained for tartrate-resistant acid phosphatase (TRAP) using a TRAP/ALP Stain Kit (FUJIFILM), according to the manufacturer’s instructions. Multinucleated TRAP-positive cells that formed on the alveolar bone surface around the second M were counted as active osteoclasts. For *in vitro* studies, RANKL- or PBS-treated human peripheral blood-derived mononuclear cells (hPBMCs) and RAW 264.7 cells were stained with TRAP/ALP Stain Kit. TRAP-positive cells with more than 2 nuclei were counted as osteoclasts. Magnified images were captured using a BZ-X800 microscope (Keyence, Osaka, Japan).

### Immunofluorescent staining

Immunofluorescence staining was performed using antibodies listed in Supplementary Table S1. The paraffin-embedded mouse maxillary sections were deparaffinized with xylene and rehydrated in ethanol. Antigen activation was performed using citrate buffer (pH 6) at 95 °C for 10 min. The sections were rinsed with PBS for 5 min. The sections were blocked by incubation in 1% BSA for 30 min at room temperature. The sections were then incubated with primary antibody overnight at 4 °C under the shade. The sections were then washed with PBS and incubated with the secondary antibody for 2 h at room temperature in the shade. Visualization was done using Keyence BZ-X800. Positive cells were counted and quantified using ImageJ software.

### Cell culture

A murine macrophage cell line RAW264.7 (TIB-71™) was obtained from American Type Culture Collection (ATCC; Manassas, VA, USA). RAW264.7 cells were cultured in Dulbecco’s modified Eagle’s medium with high glucose (Nacalai Tesque) containing 10% heat-inactivated FBS (Biowest, Vieux Bourg, France) and a penicillin-streptomycin Mixed Solution (Nacalai Tesque). Human CD14^+^ PBMCs were purchased from Lonza (Basel, Switzerland) and cultured in Roswell Park Memorial Institute (RPMI) 1640 medium (Nacalai Tesque) containing 10% heat-inactivated fetal bovine serum (FBS). In this study, no human studies were conducted.

### Osteoclast differentiation

Human PBMCs were seeded in 96-well plates at a density of 5 × 10^4^ cells/mL. Cells were cultured with or without RANKL in the presence or absence of recombinant human SPOCK-1 (rhSPOCK-1; R&D Systems, Minneapolis, Minnesota, USA) for 14 days, according to a previous report (Hayashi et al., 2022). The medium was replaced with DMEM or RPMI containing rhRANKL with or without rhSPOCK-1 every 4 days. RAW264.7 cells were seeded in 96-well plates at a density of 5 × 10^4^ cells/mL. Cells were cultured with or without rhRANKL or recombinant mouse RANKL (rmRANKL; PeproTech, Cranbury, New Jersey, USA) in the presence or absence of rhSPOCK-1 for 7 days. Osteoclasts were counted and quantified as described in TRAP staining.

### Peritoneal macrophage isolation

WT and *Spock1*-Tg mice from 8 to 10-week-old were intraperitoneally injected with 2 mL of 2% thioglycolate broth (Sigma-Aldrich) in PBS. After 2 days, the cervical vertebrae of the mice were dissected, and the mice were euthanized. The abdominal skin was removed using forceps to expose the peritoneum. Ice-cold PBS (5 mL of ice-cold PBS was injected into the peritoneal cavity, the abdomen of the mouse was gently massaged for 2 min, and PBS was collected in a 5-mL syringe. After centrifugation at 4 °C at 1000 rpm for 5 min, the pelleted cells were resuspended in 5 mL of complete RPMI1640.5×10^4^ cells were cultured at 37 °C for 1 h. Unattached cells were washed with the medium. The attached macrophages were then stimulated with *E. coli* LPS for 12 and 24 h, and the supernatant and RNA were collected as described above. qPCR and enzyme-linked immunosorbent assays ELISA were performed.

### ELISA

*E. coli* LPS-stimulated TNFα and IL-1β secretion from peritoneal macrophages isolated from WT or *Spock1*-Tg mice were measured using DuoSet Ancillary Reagent Kit2 (DY008; R&D Systems), mouse TNF-alpha DuoSet ELISA (DY410-05; R&D Systems) and mouse IL-1beta/IL-1F2 DuoSet ELISA (DY401-05; R&D Systems) according to the manufacturer’s instructions. Absorbance was measured at 450 nm using a microplate reader (Bio-Rad Laboratories, Hercules, California, USA). For quantification, peritoneal macrophages after stimulation were collected using 100 µL of CytoBuster Protein Extraction Buffer (Merck) for protein extraction. Protein concentration was measured using the Quick Start Bradford Protein Assay Kit (Bio-Rad Laboratories) and 4× LeammLi Sample Buffer (Bio-Rad Laboratories) according to the manufacturer’s protocol.

### Statistical analysis

All data are presented as mean ± standard deviation (SD). Comparisons between the two groups were performed using the unpaired Student’s t-test. One-way and two-way ANOVA, followed by *post hoc* tests, were performed to compare multiple groups using GraphPad Prism8 software (GraphPad Software, San Diego, CA, USA). Statistical significance was set at *p* < 0.05.

## Results

### *Spock1*-Tg mice showed more gingival overgrowth compared to WT mice in response to experimental periodontitis

**FIGURE 1:**
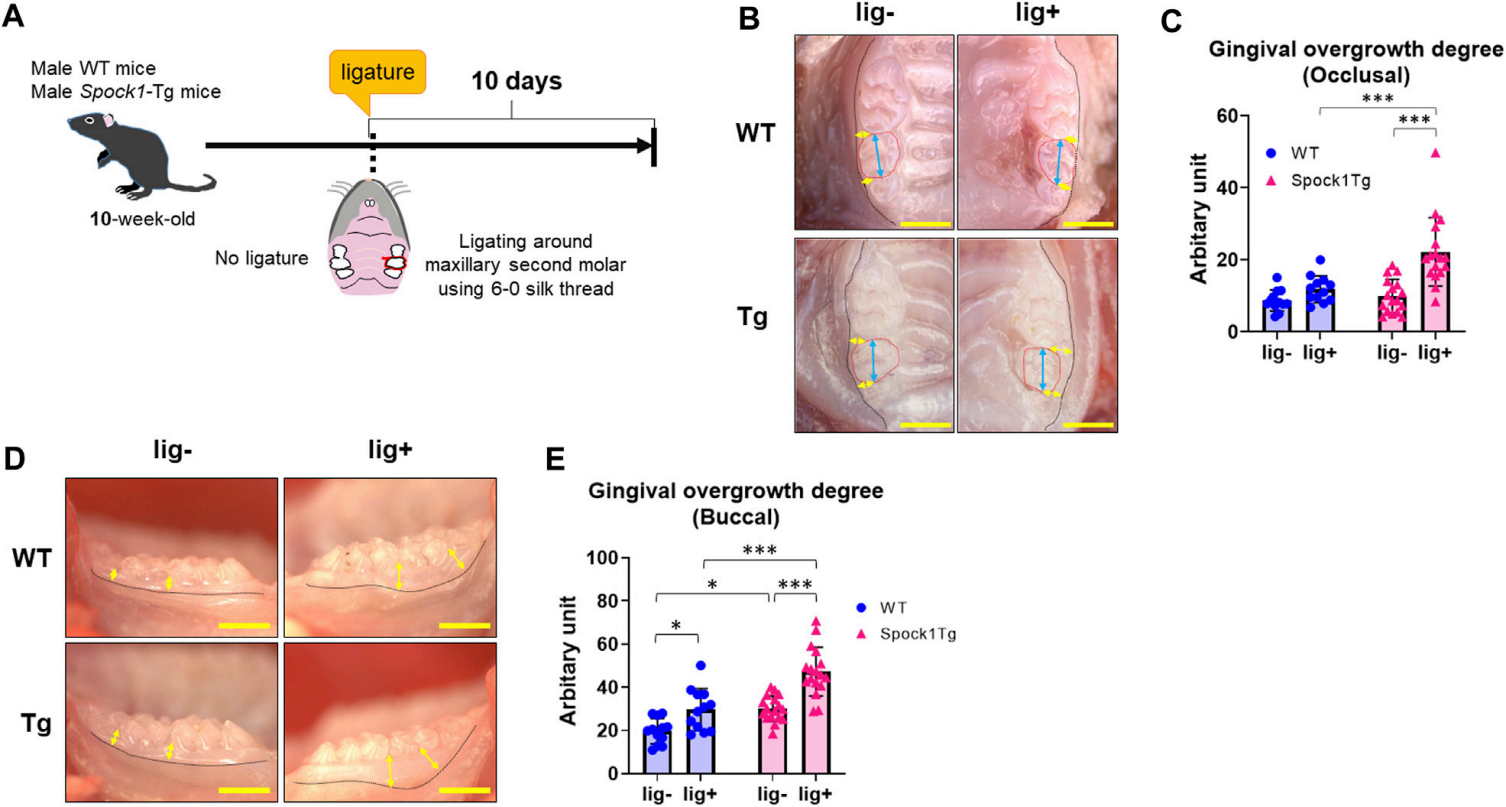
Comparison of gingival thickening induced by experimental periodontitis between WT and Spock1-Tg mice. **(A)** A schema of the experiment. **(B)** The representative photos of gingival thickening in the occlusal view in WT and Spock1-Tg mice with or without ligature-induced periodontitis. Blue double arrows indicate the width of crown and yellow double arrows indicate the measuring points for occlusal gingival thickening. Red dot lines specify secondary molars in the maxilla. **(C)** Occlusal gingival overgrowth degrees in WT and Spock-1 Tg mice with or without ligatures. **(D)** The representative photos of gingival thickening in the buccal view in WT and Spock1-Tg mice with or without ligature-induced periodontitis. Yellow double arrows indicate the measuring points for occlusal gingival thickening. Black dot lines showed the mucosa-gingival junction. **(E)** Buccal gingival overgrowth degrees in WT and Spock-1 Tg mice with or without ligatures. Scale bars = 1 mm. *p < 0.05, **p < 0.01, ***p < 0.001; n = 12–17. All individual data are shown in a scatter plot. Mean ± standard deviation (SD) is shown.

We previously reported that *Spock1*-Tg mice exhibit gingival overgrowth in the absence of periodontal inflammation due to the promotion of epithelial-to-mesenchymal transition by SPOCK-1 overexpression (Alshargabi et al., 2020). Generally, gingival or periodontal inflammation promotes gingival thickening in patients with DIGO (Sousa et al., 2011; Okanobu et al., 2017; Hatano et al., 2021). To confirm whether periodontal inflammation enhanced gingival overgrowth related to SPOCK-1 overexpression, *Spock1*-Tg and WT mice were subjected to experimental periodontitis via placement of a silk ligature (Figure 1A). The degree of occlusal overgrowth was increased by experimental periodontitis in both groups of mice (Figures 1B, C). Notably, *Spock1*-Tg mice exhibited significantly advanced occlusal gingival overgrowth compared with WT mice (Figures 1B, C). On the buccal side, periodontitis-induced gingival overgrowth in WT mice was comparable to that of *Spock1*-Tg mice without ligature (Figures 1D, E). In addition, the degree of gingival overgrowth on the buccal side of the ligatured *Spock1*-Tg mice was significantly higher than that of the ligatured WT mice (Figures 1D, E). These results suggest that gingival overgrowth was enhanced by ligature-induced experimental periodontitis in *Spock1*-Tg mice.

### Ligatured *Spock1*-Tg mice exhibited more alveolar bone loss compared to ligatured WT mice

**FIGURE 2:**
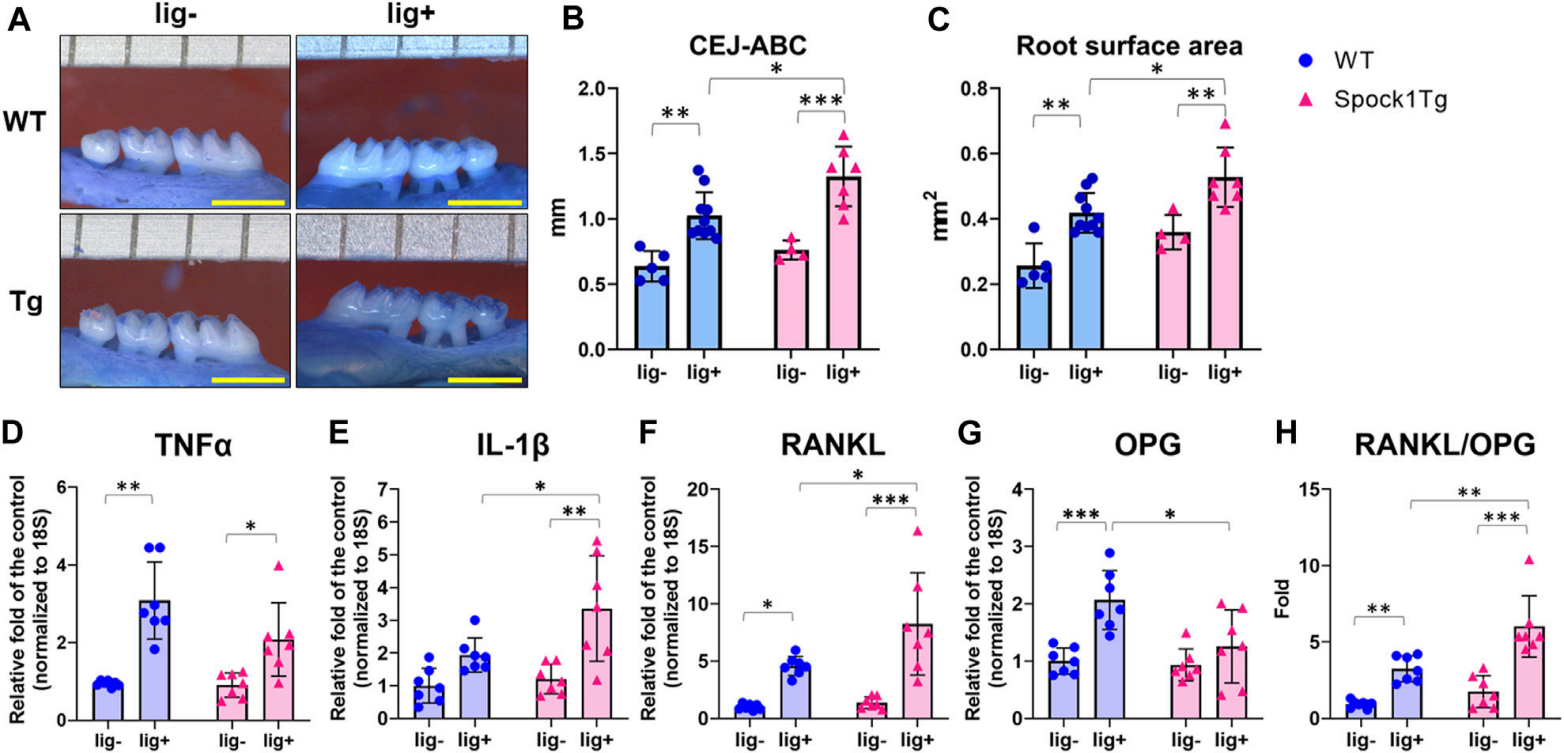
Characterization of ligature-induced periodontitis in WT and Spock1-Tg mice. **(A)** The representative photos of alveolar bone loss of the buccal view in WT and Spock1-Tg mice with or without ligature-induced periodontitis. **(B)** The sum of the distances from cemento-enamel junction to alveolar bone crest in WT and Spock1-Tg mice with or without ligatures. **(C)** The sum of the root surface area in maxillary molars in WT and Spock1-Tg mice with or without ligatures. **(D)** Tnfα, **(E)** Il-1β, **(F)** Rankl, **(G)** Opg mRNA expression levels and **(H)** Rankl-Opg mRNA expression ratio in the gingiva of WT and Spock1-Tg mice with or without ligatures. Scale bar = 1 mm. *p < 0.05, **p < 0.01, ***p < 0.001; n = 4–7. All individual data are shown in a scatter plot. Mean ± SD is shown.

Deeper periodontal pockets may contribute to the progression of periodontitis in patients with DIGO owing to the accumulation of dental plaque and subsequent periodontal inflammation. We assessed alveolar bone loss in *Spock1*-Tg and WT mice with or without ligatures. The sums of the cemantoenamel junction (CEJ)-alveolar bone crest (ABC) distance or root surface area of the maxillary molars were not significantly different between *Spock1*-Tg and WT mice under non-ligation (Figures 2A–C). In contrast, ligatured *Spock1*-Tg mice showed greater alveolar bone loss than ligatured WT mice (Figures 2A–C). Quantitative real-time polymerase chain reaction (qPCR) showed that gingival *Tnfα* expression in ligated *Spock1*-Tg mice was lower than that in ligated WT mice while gingival *Il-1β* expression was significantly elevated in ligated *Spock1*-Tg mice compared to that in ligated WT mice (Figures 2D, E). Consistent with the progression of alveolar bone loss, *Rankl* and *Opg* (osteoprogerin) mRNA expression in the gingiva of ligatured *Spock1*-Tg mice was significantly upregulated and downregulated, respectively, compared to that in ligatured WT mice (Figures 2F, G), resulting in a significant elevation of the *Rankl-Opg* expression ratio in *Spock1*-Tg mice with ligature compared to that in WT mice with ligature (Figure 2H). These results suggest that *Spock1*-Tg mice exhibited more ligature-induced alveolar bone loss resulting from dysregulation of inflammation and increased osteoclastogenesis compared to WT mice.

### CsA-treated Spock1-Tg mice with experimental periodontitis showed enhanced gingival overgrowth

**FIGURE 3:**
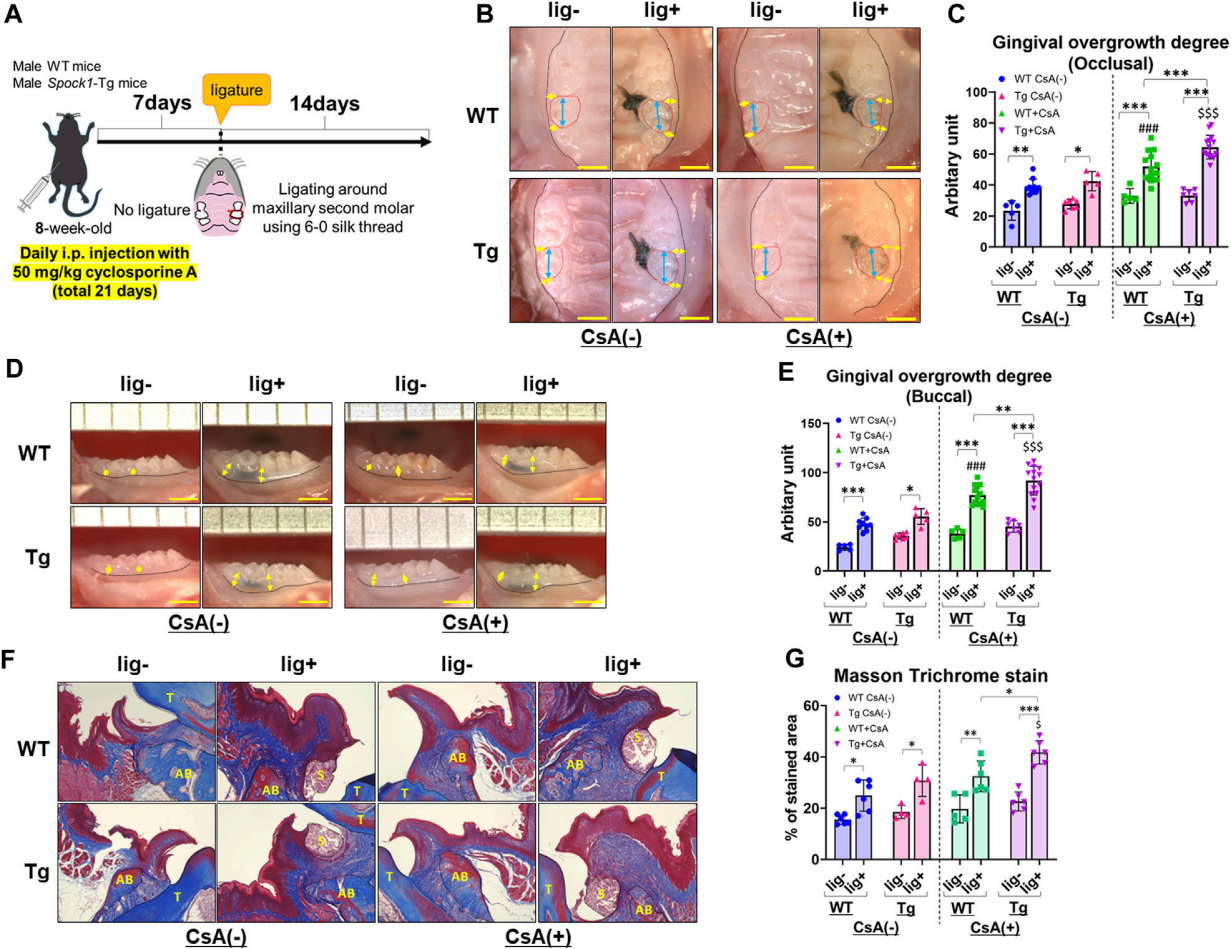
Comparison of gingival overgrowth among WT and Spock1-Tg mice with or without cyclosporin-A and/or ligature-induced periodontitis. **(A)** A scheme of the study design. **(B)** Representative photos of gingival overgrowth from occlusal view in WT and Spock1-Tg mice with or without CsA and/or experimental periodontitis. **(C)** Occlusal gingival overgrowth degree in WT and Spock1-Tg mice with or without CsA and/or experimental periodontitis. **(D)** Representative photos of gingival overgrowth from buccal view in WT and Spock1-Tg mice with or without CsA and/or experimental periodontitis. **(E)** Buccal gingival overgrowth degree in WT and Spock1-Tg mice with or without CsA and/or experimental periodontitis. **(F)** Representative photos of masson trichrome stain in the maxillae of WT and Spock1-Tg mice with or without CsA and/or experimental periodontitis. T: tooth, AB: alveolar bone, S: silk suture. **(G)** The stained area of masson trichrome stain in WT and Spock1-Tg mice with or without CsA and/or experimental periodontitis. Scale bar = 1 mm. *p < 0.05, **p < 0.01, ***p < 0.001; ###: *p* < 0.001 vs. WT lig + CsA (−); $: *p* < 0.05, $$$: *p* < 0.001 vs. Spock1-Tg lig + CsA (−). N = 4–14. All individual data are shown in a scatter plot. Mean ± SD is shown.

To investigate whether SPOCK-1 overexpression enhanced gingival overgrowth in the DIGO rodent model, we induced experimental periodontitis in both *Spock1*-Tg and WT mice by CsA treatment (Figure 3A) and assessed gingival overgrowth in these mice. As previously reported (Okanobu et al., 2017), CsA-treated WT and *Spock1*-Tg mice showed distinct gingival overgrowth on the occlusal and buccal sides in response to ligature-induced periodontitis compared to ligature-treated WT and *Spock1*-Tg mice without CsA treatment (Figures 3B–E). In addition, we found that the degree of occlusal and buccal gingival overgrowth in CsA-treated *Spock1*-Tg mice with ligature was significantly higher than that in CsA-treated WT mice with ligature (Figures 3B–E). We assessed gingival fibrosis in these mice using Masson’s Trichrome staining which revealed that the fibrotic area in the periodontal tissue. Gingival fibrosis was enhnced by experimental periodontitis in both WT and *Spock1*-Tg mice without CsA administration (Figures 3F, G). The gingival fibrosis area tended to increase in ligatured WT mice treated with CsA compared to that in ligatured WT mice without CsA treatment (Figures 3F, G). Notably, experimental periodontitis-induced fibrosis was significantly enhanced in CsA-treated *Spock1*-Tg mice compared to that in CsA-untreated *Spock1*-Tg mice (Figures 3F, G), which was consistent with the gingival thickening data (Figures 3B, C). These results suggest that gingival fibrosis by experimental periodontitis was further enhanced in CsA-treated *Spock1*-Tg mice compared to CsA-treated WT mice.

### Characterization of EMT in the gingiva of ligatured and un-ligatured WT and Spock1-Tg mice with or without CsA-administration

**FIGURE 4:**
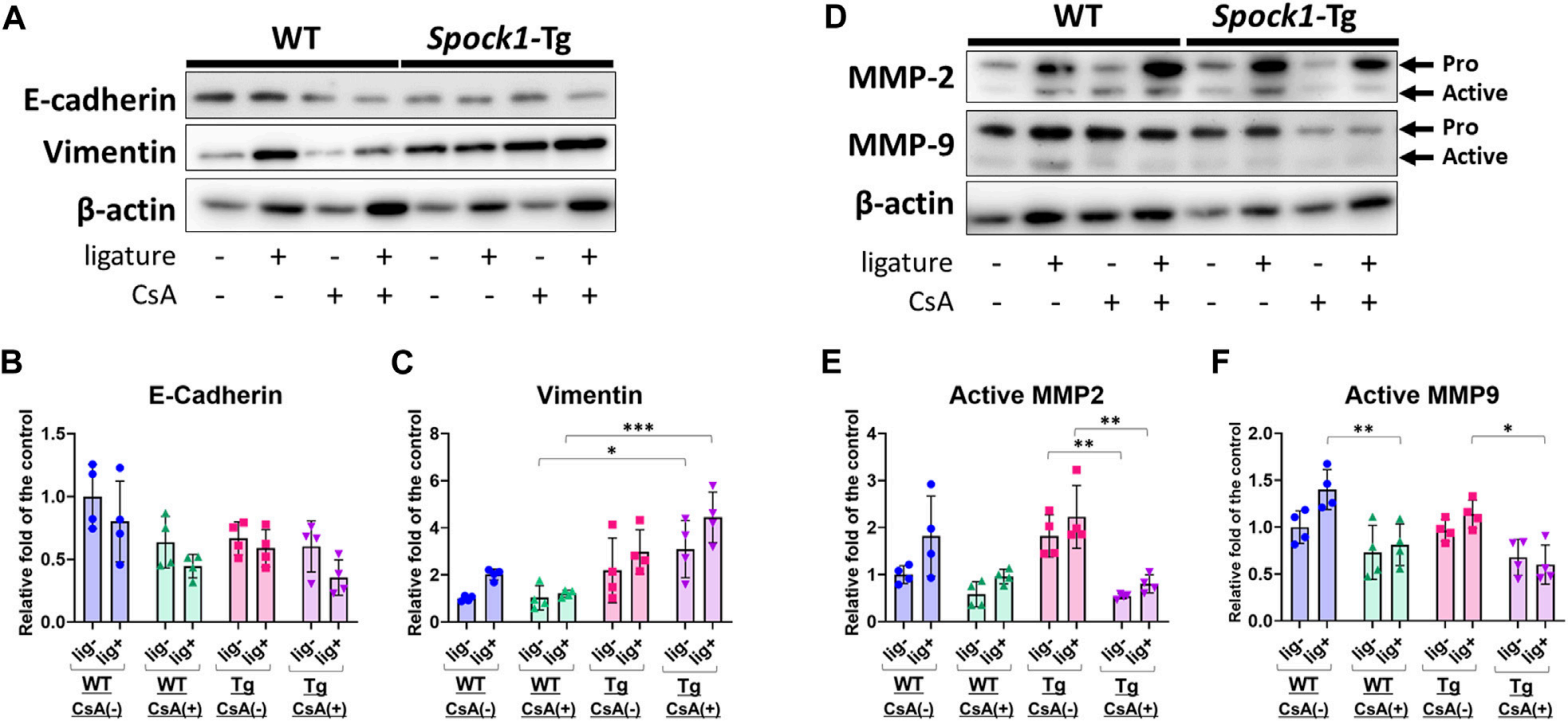
Characterization of EMT in the gingiva of ligatured and un-ligatured WT and Spock1-Tg mice with or without CsA-administration. **(A)** The representative photos of blots of E-cadherin and Vimentin in the gingiva of ligated or un-ligatured sites of WT and Spock1-Tg mice with or without CsA administration. **(B)** The quantified data of E-cadherin and **(C)** Vimentin expression levels in the gingiva of each mice groups. **(D)** The representative photos of blots of MMP-2 and MMP-9 in the gingiva of ligated or un-ligatured sites of WT and Spock1-Tg mice with or without CsA administration. **(E)** The quantified data of active-MMP-2 and **(F)** active-MMP-9 expression levels in the gingiva of each mice groups. *: *p* < 0.05, **: *p* < 0.01, ***: *p* < 0.001. N = 4. All individual data are shown in a scatter plot. Mean ± SD is shown.

Next, we detected EMT-related molecules to confirm the EMT characterization in the gingiva of ligatured and un-ligatured WT and *Spock1*-Tg mice in the presence or absence of CsA administration and/or experimental periodontitis. CsA-treated WT mice showed downregulation of E-cadherin and no change in Vimentin in the gingiva (Figures 4A–C), which is consistent with previous finding in humans (Chen et al., 2023). Induction of periodontitis tend to decrease E-cadherin and increase Vimentin in both WT and CsA-treated WT mice. *Spock1*-Tg mice exhibited downregulation of E-cadherin and upregulation of Vimentin as previously reported (Alshargabi et al., 2020), and CsA administration significantly increased Vimentin level in the gingiva of *Spock1*-Tg mice compared with that of WT mice (Figures 4A–C). The combination of CsA and experimental periodontitis resulted in the lowest E-cadherin and the highest Vimentin expression in the gingiva of *Spock1*-Tg mice among all of the mice groups (Figures 4A–C). Both MMP-2 and 9 protein levels were not significantly changed in the gingiva of un-ligatured site from WT and *Spock1*-Tg mice with or without CsA, while MMP-2 tended to be increased in the gingiva of ligated site from WT and *Spock1*-Tg mice with or without CsA administration and gingival MMP-9 levels were significantly downregulated in ligatured WT and *Spock1*-Tg mice (Supplementary Figures S2A, S2B). In contrast, MMP-2 and 9 activities were decreased by administration of CsA despite of concomitant induction of experimental periodontitis (Figures 4D–F).

### Ligatured Spock1-Tg mice exhibited greater alveolar bone loss than ligatured WT mice under CsA-treatment

**FIGURE 5:**
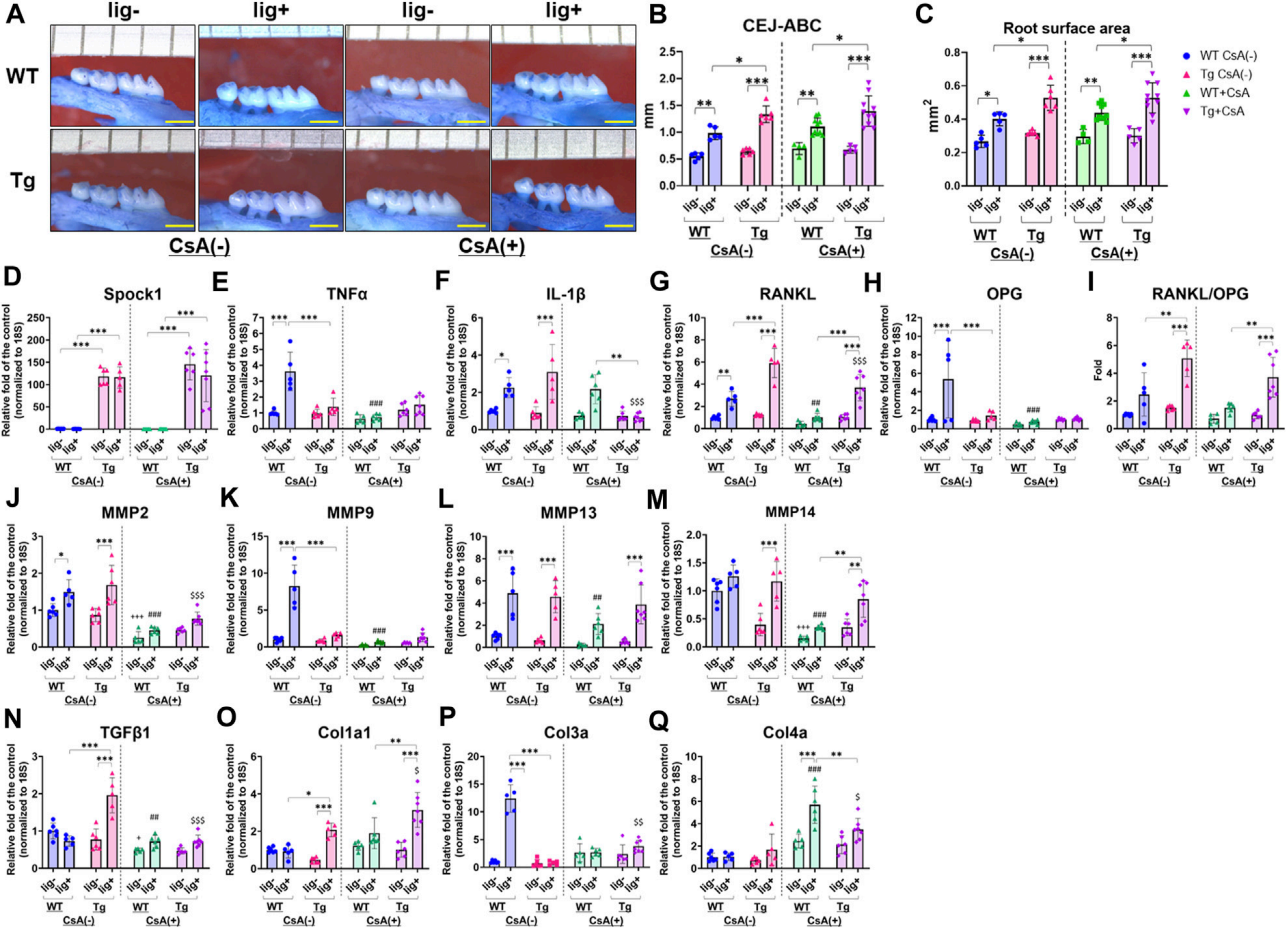
Characterization of ligature-induced periodontitis in WT and Spock1-Tg mice with or without cyclosporin-A administration. **(A)** The representative photos of alveolar bone loss of the buccal view in WT and Spock1-Tg mice with or without ligature-induced periodontitis and/or CsA treatment. **(B)** The sum of the distances from cemento-enamel junction to alveolar bone crest and **(C)** the sum of the root surface area in maxillary molars in WT and Spock1-Tg mice with or without ligatures and/or CsA treatment. **(D)** Spock1, **(E)** Tnfα, **(F)** Il-1β, **(G)** Rankl, **(H)** Opg mRNA expression levels and **(I)** Rankl-Opg mRNA expression ratio in the gingiva of WT and Spock1-Tg mice with or without ligatures. **(J)** Mmp2, **(K)** Mmp9, **(L)** Mmp13, **(M)** Mmp14, **(N)** Tgfβ1, **(O)** Col1a1, **(P)** Col3a and **(Q)** Col4a mRNA expression levels in the gingiva of WT and Spock1-Tg mice with or without ligatures. Scale bar = 1 mm. *: *p* < 0.05, **: *p* < 0.01, ***: *p* < 0.001; +: *p* < 0.05, +++: *p* < 0.001 vs. WT lig- CsA (−); ##: *p* < 0.01, ###: *p* < 0.001 vs. WT lig + CsA (−); $: *p* < 0.05, $$: *p* < 0.01, $$$: *p* < 0.001 vs. Spock1-Tg lig + CsA (−). N = 4–10. All individual data are shown in a scatter plot. Mean ± SD is shown.

Next, we assessed the alveolar bone loss in *Spock1*-Tg and WT mice with combination of the presence or absence of ligatures and CsA. There were no differences in the alveolar bone levels between CsA-treated and untreated mice without ligatures (Figures 5A–C). In contrast, ligatured *Spock1*-Tg mice showed significantly greater alveolar bone loss than ligatured WT mice treated with CsA-treatment (Figures 5A–C). Additionally, we found that the extent of alveolar bone loss in ligatured *Spock1*-Tg and WT mice treated with CsA was similar to that in untreated *Spock1*-Tg and WT mice (Figures 5A–C). These results suggest that CsA treatment did not affect the extent of alveolar bone loss induced by ligature in both *Spock1*-Tg and WT mice despite immunosuppressive effect of CsA.

### Characterization of periodontitis in CsA-treated Spock1-Tg mice with ligatures

CsA suppressed inflammatory responses in immune cells by inhibiting calcium-mediated activation (Marx et al., 1990; Grinyo et al., 2004; Kobayashi et al., 2007; Medyouf et al., 2007; Matsuda et al., 2019). We then measured periodontitis-related gene expression in the gingiva of CsA-treated or untreated *Spock1*-Tg and WT mice, with or without ligatures. First, *Spock1* expression in the gingiva of *Spock1*-Tg mice was increased by approximately 100-fold compared to the gingiva of WT mice (Figure 5D). Neither ligation nor CsA treatment affected *Spock1* expression in the gingiva of both WT and *Spock1*-Tg mice (Figure 5D). In the untreated condition, ligation clearly induced *Tnfα* expression in WT mice gingiva, and *Spock1*-Tg mice with ligature showed significantly lower *Tnfα* expression in the gingiva than WT mice with ligature (Figure 5E). In contrast, CsA treatment significantly suppressed ligature-induced *Tnfα* expression in WT mouse gingiva (Figure 5E). Ligature-induced gingival *Il-1β* expression was comparable between *Spock1*-Tg and WT mice without CsA treatment but was significantly suppressed in *Spock1*-Tg mice gingiva but not in WT mice gingiva with CsA treatment (Figure 5F). Gingival *Rankl* expression in ligatured *Spock1*-Tg mice was significantly higher than that in ligatured WT mice, with or without CsA treatment (Figure 5G), and CsA treatment significantly suppressed gingival *Rankl* expression in both WT and *Spock1*-Tg mice (Figure 5G). However, *Opg* expression in the gingiva of *Spock1*-Tg mice was significantly lower than that in the gingiva of WT mice, resulting in a significantly increased *Rankl-Opg* expression ratio in *Spock1*-Tg mice gingiva compared to WT mice gingiva (Figures 5H, I).

Among the Matrix metalloproteinases (MMPs), gingival *Mmp2* expression was significantly upregulated by experimental periodontitis in both WT and *Spock1*-Tg mice (Figure 5J). *Mmp9* expression was significantly lower in ligatured *Spock1*-Tg mouse gingiva than in ligatured WT mouse gingiva, but *Mmp2*, *13*, and *14* expressions did not change (Figures 5J–M). CsA treatment suppressed ligature-induced *Mmp2*, *9*, *13*, and *14* expressions in WT mouse gingiva (Figures 5J–M). In contrast, *Mmp2*, but not *Mmp9*, *13*, and *14*, expression was suppressed by CsA treatment (Figures 5J–M). Ligature-induced gingival *Mmp13* and *14* expressions in CsA-treated *Spock1*-Tg mice was upregulated compared with that in CsA-treated WT mice (Figures 5L, M).

The acceleration of collagen production is a significant feature of DIGO. The expression of *Tgfβ1*, a cytokine related to fibrosis, was significantly elevated by ligature in *Spock1*-Tg mice without CsA treatment, but CsA treatment suppressed its expression in *Spock1*-Tg mice (Figure 5N). We also found that ligature-induced *Col1a*, but not *Col3a* and *Col4a*, expression was increased in *Spock1*-Tg mice compared to WT mice with or without CsA treatment, and CsA treatment significantly enhanced *Col1a* expression in the gingiva of *Spock1*-Tg mice (Figures 5O–Q).

The expression of *Il-2* tended to be downregulated in *Spock1*-Tg mice at basal and increased by experimental periodontitis in both WT and *Spock-1*Tg mice. As previous report that CsA inhibited *Il-2* expression in T-cells (Nishiyama et al., 2004; Kobayashi et al., 2007), CsA administration significantly inhibited *Il-2* expression in both WT and *Spock1*-Tg mice nevertheless with or without ligatures (Supplementary Figure S2).

These results suggest that experimental periodontitis-mediated osteoclastogenesis and collagen accumulation were enhanced by CsA administration in *Spock1*-Tg mice despite suppression of inflammatory and MMPs gene expressions.

### Characterization of neutrophil recruitment and osteoclastogenesis in ligatured *Spock1*-Tg and WT mice with or without CsA-treatment

**FIGURE 6:**
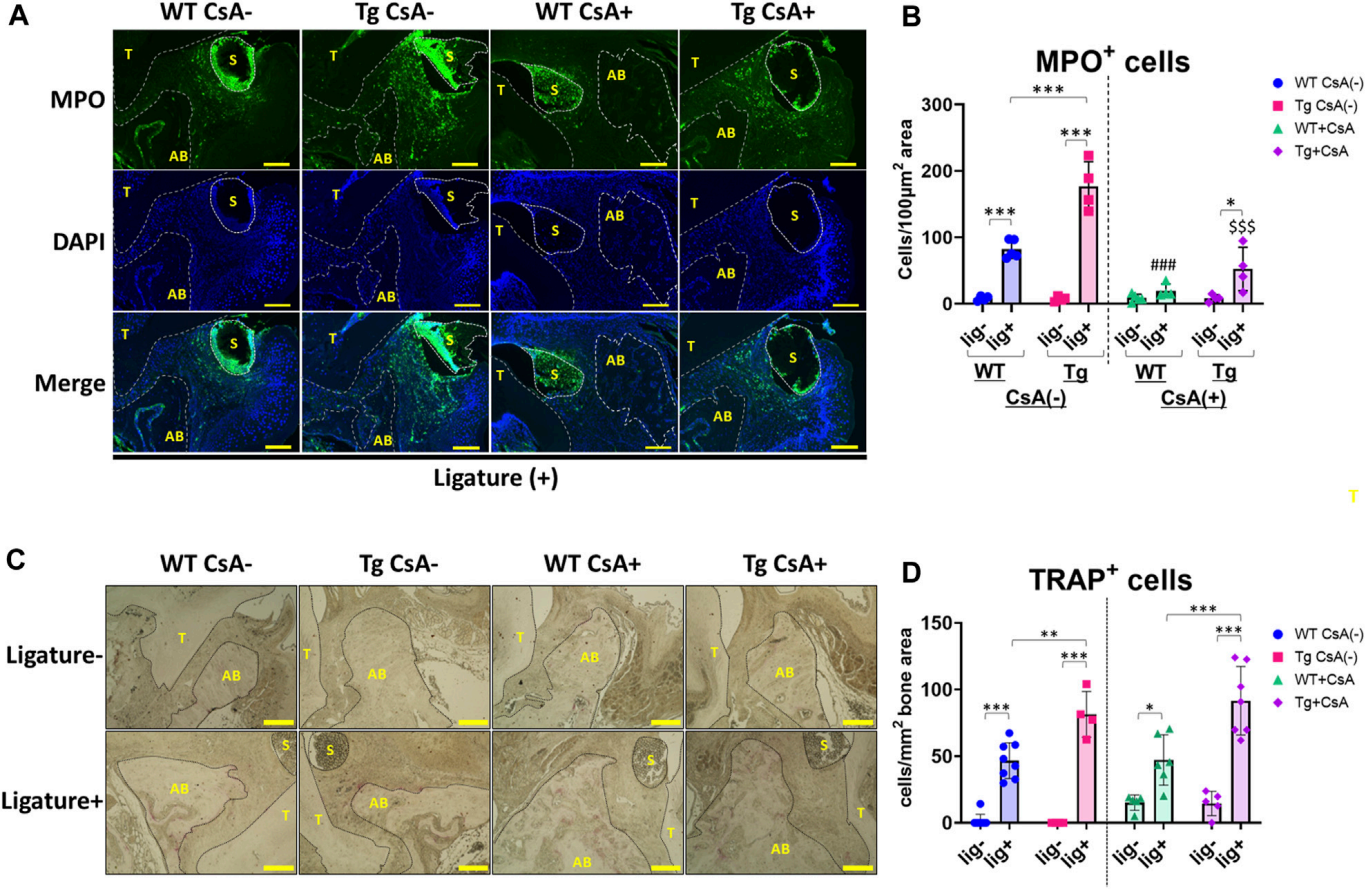
Histological analyses in the periodontal tissue of ligatured WT and Spock1-Tg mice with or without CsA treatment. **(A)** Representative photos of immunofluorescent stain against Myeloperoxidase (MPO) in the periodontal tissue of ligatured WT and Spock1-Tg mice with or without CsA administration. **(B)** MPO-positive cells in the periodontal tissue of ligatured WT and Spock1-Tg mice with or without CsA administration. **(C)** Representative photos of TRAP stain in the periodontal tissue of ligatured WT and Spock1-Tg mice with or without CsA administration. **(D)** TRAP-positive cells in the periodontal tissue of ligatured WT and Spock1-Tg mice with or without CsA administration. Scale bar = 100 µm. *: *p* < 0.05, **: *p* < 0.01, ***: *p* < 0.001; ###: *p* < 0.001 vs. WT lig + CsA (−); $$$: *p* < 0.001 vs. Spock1-Tg lig + CsA (−). N = 4–9. T: tooth, AB: alveolar bone, S: silk suture. All individual data are shown in a scatter plot. Mean ± SD is shown.

Dysregulation of neutrophil infiltration may contribute to the exacerbation of alveolar bone loss (Van Dyke et al., 1980; Kim et al., 2020; Shinjo et al., 2023). To assess neutrophil infiltration in the periodontal tissue, we performed immunofluorescence staining to detect neutrophils in periodontal tissues of WT and *Spock1*-Tg mice. Immunofluorescent staining for myeloperoxidase (MPO) showed significantly increased ligature-induced neutrophil infiltration in the periodontal tissue of CsA-untreated *Spock1*-Tg mice compared to that in CsA-untreated WT mice (Figures 6A, B). CsA administration significantly suppressed neutrophil infiltration in the periodontal tissue of both ligatured *Spock1*-Tg and WT mice, but MPO-positive cells in the gingiva were still increased in ligatured *Spock1*-Tg mice treated with CsA compared to WT mice treated with CsA (Figures 6A, B).

Consistent with *Rankl* expression data, ligatured *Spock1*-Tg mice showed a significantly increased number of TRAP-positive cells in the alveolar bone compared to ligatured WT mice (Figures 6C, D). CsA treatment did not affect the number of osteoclasts in both *Spock1*-Tg and WT mice (Figures 6C, D). These results suggest that *Spock1*-Tg mice exhibited the dysregulation in neutrophil recruitment and enhancement of osteoclastogenesis in response to ligatures under both presence and absence of CsA administration.

### SPOCK1 promoted the differentiation of osteoclast in murine macrophages and human PBMCs

**FIGURE 7:**
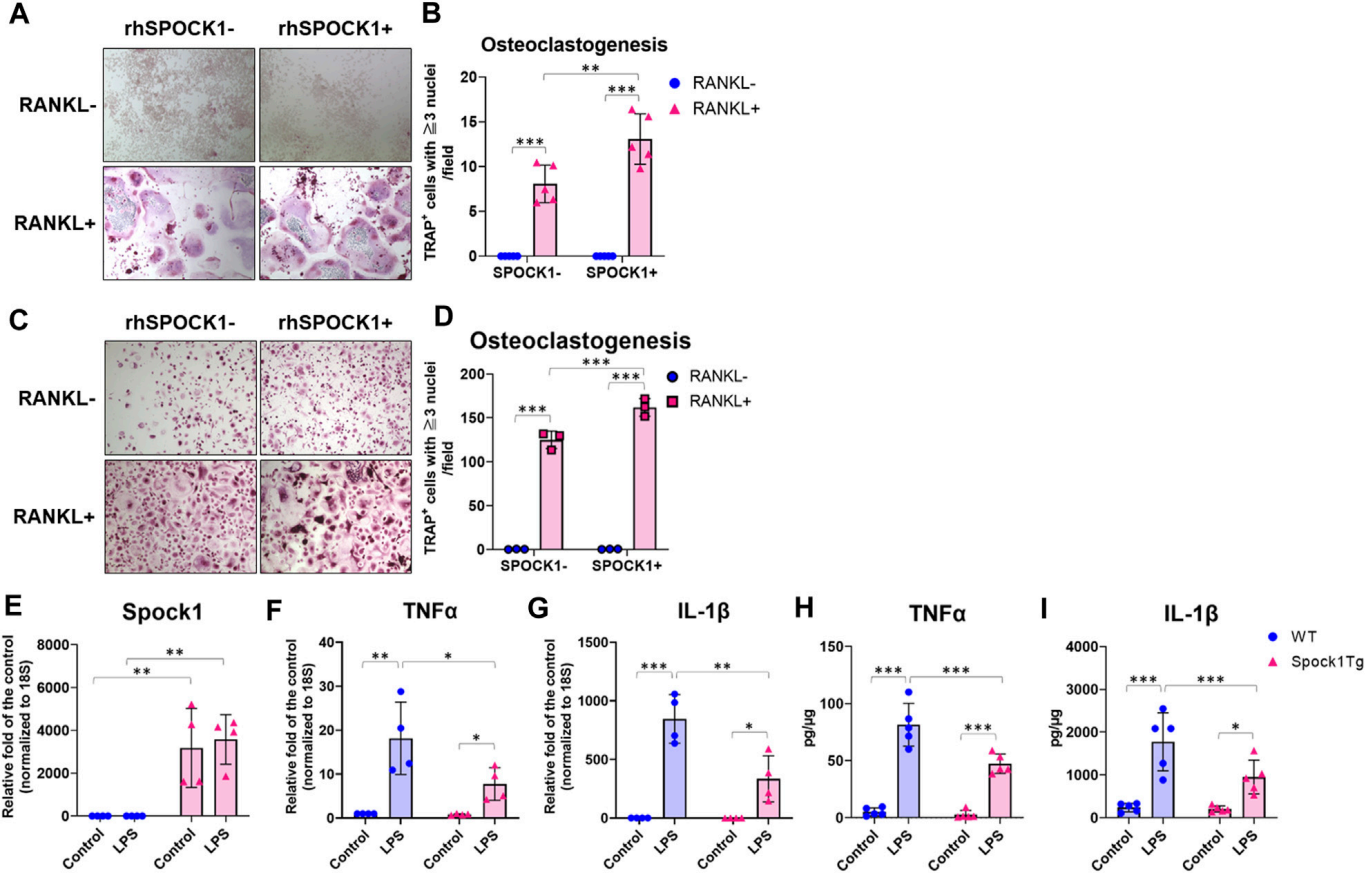
Effect of SPOCK1 on the osteoclast differentiation and inflammatory response in macrophages. **(A)** Representative photos of RANKL- or PBS-treated hPBMC with or without co-incubation with rhSPOCK1. **(B)** TRAP-positive cells in each condition of hPBMC. **(C)** Representative photos of RANKL- or PBS-treated RAW264.7 with or without co-incubation with rhSPOCK1. **(D)** TRAP-positive cells in each condition of RAW264.7. **(E)** Spock1, **(F)** Tnfα and **(G)** Il-1β mRNA expression in peritoneal macrophages from WT and Spock1-Tg mice in the presence or absence of *Escherichia coli* LPS. **(H)** TNFα and **(I)** IL-1β secretion from peritoneal macrophages from WT and Spock1-Tg mice in the presence or absence of *Escherichia coli* LPS. *: *p* < 0.05, **: *p* < 0.01, ***: *p* < 0.001. N = 3–5. All individual data are shown in a scatter plot. Mean ± SD is shown.

To confirm whether SPOCK-1 positively regulates osteoclast differentiation, we compared osteoclastogenesis in the RAW264.7 and human peripheral blood-derived mononuclear cells (PBMC) with or without SPOCK-1. SPOCK-1 significantly increased TRAP-positive RAW264.7 cells after incubation with RANKL but did not induce osteoclastogenesis in RAW264.7 cells by itself (Figures 7A, B). In human PBMCs, SPOCK-1 promoted RANKL-induced osteoclastogenesis, but did not induce osteoclast differentiation (Figures 7C, D). These results suggest that SPOCK-1 promoted the RANKL-mediated differentiation of osteoclasts in both murine macrophages and human PBMCs.

### *Spock1*-Tg mice macrophages showed less expression and production of TNFα and IL-1β expression in response to LPS.

We measured the inflammatory response of macrophages isolated from *Spock1*-Tg and WT mice. *Spock1* mRNA expression in peritoneal macrophages of *Spock1*-Tg mice was significantly higher than that in macrophages of WT mice (Figure 7E). Peritoneal macrophages from *Spock1*-Tg mice also showed significantly lower expression of *Tnfα* and *Il-1β* in response to *Escherichia coli* LPS stimulation (Figures 7F, G). Consistently, *E. coli* LPS-induced TNFα and IL-1β production was also significantly lower in *Spock1*-Tg mice macrophages compared to WT mice macrophages (Figures 7H, I). These results suggest that macrophages from *Spock1*-Tg mice exhibited less inflammatory response to infectious stimulus.

## Discussion

In this study, we explored the contribution of factors such as EMT, collagen production and inflammation on the onset of DIGO. We accomplished this by using a silk ligature model of periodontitis and the transgenic *Spock1*-Tg mice that induce the DIGO phenotype by facilitating EMT. Moreover, we administered CsA, which induces DIGO through a different mechanism. By implementing various combinations of these factors, we were able to explore their individual and combined contributions to DIGO onset and thus effectively evaluate the effect of multiple factors on DIGO pathogenesis.

The pathophysiological feature of human DIGO has been reported to be different depending on the causative agents, especially regarding fibrosis and inflammation, while thickening of both the epithelium and connective tissue, and elongation of the rete peg deep into the connective tissue could be observed in these cases (Van Dyke et al., 1980; Hallmon and Rossmann, 1999; Kim et al., 2020). A comparative study has revealed that CsA-related DIGO mainly displayed inflammation with a little fibrotic area limited in the sulcular epithelium and subepithelial connective tissue, and phenytoin-related DIGO exhibited substantial fibrosis without inflammation conversely, and nifedipine-related DIGO showed the mixed features of CsA and phenytoin (Uzel et al., 2001). Thus, ideally, an animal model of the disease must fulfill criteria specific to the causative agent.

Certain congenital systemic diseases that accompany DIGO-like phenotypes are good models for understanding DIGO pathogenesis. I-cell disease, a lysosomal storage disease, is one such condition. In I-cell diseases, several lysosomal enzyme activities are impaired owing to disorders in the maturation processes of these enzymes (Kose et al., 2019). Therefore, we initially hypothesized that one or more of the enzymes whose activity was impaired in these subjects might be responsible for the pathogenesis of DIGO and focused on cathepsin L; this is because *in vitro* experiments demonstrated that CsA appeared to suppress cathepsin-L gene expression. Furthermore, cathepsin L-null mice exhibit a DIGO-like phenotype (Nishimura et al., 2002). However, I-cell disease and cathepsin-L-null mice not only showed a DIGO-like phenotype but also several systemic phenotypic changes, which are not all usually observed in human DIGO. We thus considered that causative drugs may not necessarily directly suppress cathepsin-L activity, and it is possible that other factors influenced by the causative drug partially contributed to the downregulation of cathepsin L activity. Therefore, we focused on SPOCK-1, which has been reported to suppress cathepsin L activity along with other enzymes (Bocock et al., 2003). Based on this hypothesis, we generated SPOCK-1 transgenic mice. These mice exhibited DIGO-like gingival thickening as demonstrated in our previous study (Alshargabi et al., 2020). More importantly, SPOCK-1 is reported to be involved in the EMT process, and elongated rete pegs, a typical feature of DIGO, have been observed in *Spock1*-Tg mice. However, as mentioned previously, gingival thickening is limited in these mice and does not cover the entire crown of the tooth, as is sometimes observed in humans.

Plaque control is recommended for the treatment of DIGO. Most importantly, CsA, which is reported to induce frequent DIGO in human and mouse models (Okanobu et al., 2017; Hatano et al., 2021), is known to inhibit CN/NF-AT signals. Therefore, we hypothesized that other factors that promote EMT cooperatively contributed to the development of DIGO and focused on the involvement of inflammation and NF-AT signaling.

In this study, we show that ligatured *Spock1*-Tg mice showed greater gingival overgrowth than ligatured WT mice (Figures 1A–E). These data suggested that periodontal inflammation and SPOCK-1 cooperatively enhanced gingival thickening. We also found that ligature-induced alveolar bone loss was greater in *Spock1*-Tg mice than in WT mice (Figures 2A–C). *Tnfα *expression was downregulated but *IL-1β* expression was upregulated in the gingiva of ligatured *Spock1*-Tg mice compared to that of ligatured WT mice (Figures 2D, E), suggesting that *Spock1*-Tg mice exhibited partial suppression in inflammatory response to periodontal infection. So far, no reports showed the direct interaction among IL-1β and MMP-2, 9 and NF-AT, related to EMT molecules, suggesting that IL-1β may contribute to not progression of gingival overgrowth but acceleration of alveolar bone loss via promoting osteoclast differentiation (Kim et al., 2009). Notably, gingival *Rankl* expression was upregulated in ligatured *Spock1*-Tg mice compared to that in ligatured WT mice, which was consistent with increased alveolar bone loss and osteoclastogenesis in the alveolar bone (Figures 2F–H; Figures 6C, D). Interestingly, histological analyses showed that ligature-induced experimental periodontitis caused gingival fibrosis, was markedly accelerated by CsA administration (Figures 3F,G). *Spock1*-Tg mice showed an increased fibrotic area in the gingival connective tissue in response to ligature-induced periodontitis compared to WT mice, whereas the extent of epithelial thickness in *Spock1*-Tg mice with periodontitis was comparable to that of WT mice with periodontitis (Figures 3F, G). Under CsA treatment, ligatured WT mice exhibited an increase in the fibrotic area in the gingival connective tissue, whereas ligatured *Spock1*-Tg mice showed a much larger fibrotic area in the gingival connective tissue and thickening of the gingival epithelium (Figures 3F, G). Elongation of the pete pegs with widening was observed in CsA-treated mice with experimental periodontitis, which is consistent with human gingival tissue with CsA-mediated DIGO (Bullon et al., 2007; Chen et al., 2023).

*Spock1*-Tg has been reported to show downregulation of E-cadherin and upregulation of Vimentin in the gingival epithelium, indicating that EMT-like phenotype (Alshargabi et al., 2020). Patients treated with CsA showed downregulation of E-cadherin but no change in Vimentin (Chen et al., 2023), implying CsA might cause partial EMT. WB data consistently showed CsA administration induced decreased E-cadherin and no change in Vimentin in mice (Figures 4A–C). Experimental periodontitis could induce small downregulation of E-cadherin and upregulation of Vimentin (Figures 4A–C). Combination of CsA and experimental periodontitis seemed to additively downregulate E-cadherin but not in Vimentin, suggesting CsA-mediated downregulation of Vimentin might be stronger than periodontitis-induced upregulation of Vimentin. On the other hand, CsA-treated *Spock1*-Tg mice did not show downregulation of Vimentin in the gingiva. Combination of CsA and periodontitis exhibited additive suppression of E-cadherin and upregulation of Vimentin in *Spock1*-Tg mice. Taken together, we considered that CsA-related EMT could be partially different from SPOCK1-related EMT in gingiva, but both contribute to gingival overgrowth under periodontitis. Furthermore, CsA and SPOCK1-related EMT could enhance gingival overgrowth under periodontitis in additive manner. Although the action of CsA has been mainly studied using immune cells such as T-lymphocytes, and CN/NF-AT is ubiquitously expressed in many cell types, its role in fibroblasts has not been well studied. However, recently, the deletion of CN in fibroblasts was reported to enhance collagen expression, accompanied by abnormal cell morphology (Yamada et al., 2000). CsA significantly suppressed pro-inflammatory cytokine gene expression such as *Tnfα* and *IL-1β* in *Spock1*-Tg mice (Figures 4E,F). Pro-inflammatory cytokines are well-known inducers of matrix-degrading enzymes in connective tissue such as MMPs (Birkedal-Hansen, 1993; Lieberman et al., 2019). Therefore, it is likely that these immune cell-derived cytokines such as *Tnfα* and *IL-1β* in chronic periodontitis conditions induce matrix breakdown in the connective tissues (Richardson and Dodge, 2000; Gordon et al., 2009). It has been reported that CsA suppressed *Il-2* expression in T-cells (Nishiyama et al., 2004; Kobayashi et al., 2007), and our data consistently showed CsA administration downregulated ligature-induced *Il-2* expression in the mice (Supplementary Figure S2). *Spock1*-Tg mice tend to suppress *Il-2* expression as well, implying that immunosuppressive effect on *Il-2* similar to *Tnfα* and *Il-1β* (Figures 4E, 6F,G). However, CsA strongly suppresses MMP activity in fibroblasts owing to the reduced effects of pro-inflammatory cytokines, and this inhibition cannot catch up with the matrix turnover caused by increased collagen synthesis via impaired CN/NF-AT signaling. Therefore, the clinical observation that thorough plaque control has a favorable influence on the management of DIGO may mainly be mediated by the avoidance of the suppression of MMP activity rather than the remission of inflammation. In other words, thorough plaque control may have paradoxical effects on the recovery of matrix-degrading activity.

Nifedipine, phenytoin, and CsA could induce TGFβ1 production in gingival fibroblasts and TGFβ1 could increase SPOCK1 expression in gingival epithelial cells (Alshargabi et al., 2020). In Figure 5N, *Tgfβ1* expression was significantly upregulated in the gingiva at the ligatured side but not the un-ligatured side of *Spock1*-Tg mice compared with the gingiva at the ligatured side of WT mice. TGFβ1 is one of the potent driving factors of collagen production. A previous study has reported that MMP9 transcription was suppressed by TGFβ1 in immune cells (Ogawa et al., 2004). In contrast, another study has shown that SPOCK1 could upregulate MMP-9 expression in hepatocarcinoma cell lines (Li et al., 2013). Taking these reports into consideration, the relationship among SPOCK1, TGFβ1, and MMPs might be different depending on the cell types and disease models. The detail of the molecular basis of the complicated mechanism in the periodontitis-associated DIGO is needed to be elucidated in the future.

The present study showed that MMP-2 levels in the gingiva were upregulated in both WT and *Spock1*-Tg mice (Supplementary Figure S2A), and active-MMP-2 levels in the gingiva were upregulated in *Spock1*-Tg mice and experimental periodontitis (Figures 4D,E). CsA treatment clearly suppressed MMP-2 activity in the gingiva at both ligatured and un-ligatured sides of WT and *Spock1*-Tg mice (Figures 4D,E). Experimental periodontitis also increased MMP-9 levels in the gingiva of WT mice (Supplementary Figure S2B). CsA treatment tends to suppress MMP-9 and active-MMP-9 levels in both WT and *Spock1*-Tg mice (Figures 4D,F). These data provide speculation about the difference in mode of EMT between CsA and SPOCK1 in DIGO. The comparative investigation in detail of the complexed molecular basis of the difference between SPOCK1-related and CsA-related EMT should be conducted in future studies.

The influence of SPOCK-1 and CsA on bone remodeling remains inconclusive. SPOCK-1 itself appeared to stimulate RANKL-induced osteoclast differentiation (Figures 7A–D). However, introduction of the NF-AT variant into osteoblasts results in increased bone mass, and NF-AT is known to accelerate osteoclast differentiation (Takayanagi, 2007). In our animal models, we did not observe a remarkable increase in bone resorption following the mixed stimulation of SPOCK-1 overexpression, inflammation, and administration of CsA (Figures 4A–C). Long-term CsA administration is associated with osteopenia and decreased bone density. Our results showed that CsA treatment did not affect the induction of TRAP-positive cells on the surface of the alveolar bone in ligatured WT and *Spock1*-Tg mice (Figures 5C, D); however, *Spock1*-Tg mice clearly showed more alveolar bone loss (Figures 5A–C) with more infiltration of neutrophils with or without CsA treatment (Figures 6A, B), suggesting that SPOCK-1 mediated enhancement in RANKL-induced osteoclastogenesis, CsA does not affect directly monocyte and neutrophil chemotaxis, monocyte phagocytosis, and neutrophil bactericidal activity (Kharazmi et al., 1985), and suppresses osteoclast differentiation. However, the present study showed the number of TRAP-positive cells was not affected by CsA administration under periodontitis (Figures 6C, D). One possible explanation would be the suppression of *Opg*, counteracts against RANKL, expression by CsA (Figure 5H), resulting in compatible osteoclastogenesis with no CsA treatment despite significant downregulation of *Rankl* by CsA (Figure 5G). Taken together, CsA-induced reduction in alveolar bone density, and increased neutrophils due to partial suppression of infectious inflammation cooperatively contribute to the progression of periodontitis. Although the data showed SPOCK-1 could enhance osteoclast differentiation in the current study (Figures 7A–D), the precise mechanism of how SPOCK-1 plays in osteoclastogenesis remains unclear since the receptor of SPOCK-1 has not been identified. The possible mechanism is that SPOCK-1 overexpression and knockdown could enhance and decrease NF-κB pathway activation via upregulation of phosphorylation of IκBα and p65 (Liu et al., 2021; Cui et al., 2022). These data suggest that both endogenous and exogenous SPOCK-1 could potentiate osteoclast differentiation, resulting in increased bone loss. Clinically, we sometimes observe exostosis of the alveolar bone during long-term administration of DIGO-causing drugs. Thus, DIGO could be associated with increased alveolar bone mass; however, this issue needs to be further considered at both the clinical and experimental levels.

The present study had three major limitations. First, only CsA was used to induce DIGO related to periodontitis in this study. Administration of CsA, phenytoin, and nifedipine could induce gingival overgrowth and dental plaque accumulation promoted in rat study (Nishikawa et al., 1996). It has been reported that CsA-induced gingival overgrowth pathophysiologically differs from phenytoin- and nifedipine-induced gingival overgrowth in humans; CsA-induced gingival overgrowth exhibits a high degree of inflammation with less collagen accumulation, while phenytoin-related gingival overgrowth is highly fibrotic and nifedipine-induced gingival overgrowth displays a mixed lesion (Uzel et al., 2001). So far, there have been reported DIGO model using medications such as nifedipine, phenytoin and CsA with or without periodontitis, showing some extent of gingival overgrowth at incisors and molars (Fernandes et al., 2010; Assaggaf et al., 2015). The current model using *Spock1*-Tg mice with CsA administration in combination with experimental periodontitis is not fully mimic human DIGO even though it shows highly fibrotic gingival overgrowth that almost covers the tooth crown. However, so far, it remains unclear whether the combination of phenytoin or nifedipine with ligature-induced periodontitis may induce DIGO in rodents. Future studies are required to determine whether the combination of these drugs such as phenytoin and nifedipine with experimental periodontitis might cause DIGO and the progression of periodontitis, similar to CsA. Second, we only used male mice in the study. A review article suggested that male rodents were more susceptible to nifedipine-mediated DIGO than female rodents (Nishikawa et al., 1996). Female mice should be tested if their susceptibility to DIGO is lower than male mice in the future. Third, we did not confirm the findings in human gingival samples because of the difficulty in collecting gingival samples from patients treated with CsA, as these patients are more susceptible to infection and the COVID-19 pandemic. Future clinical investigation into the association among CsA, periodontitis, and the degree of DIGO is necessary.

**FIGURE 8:**
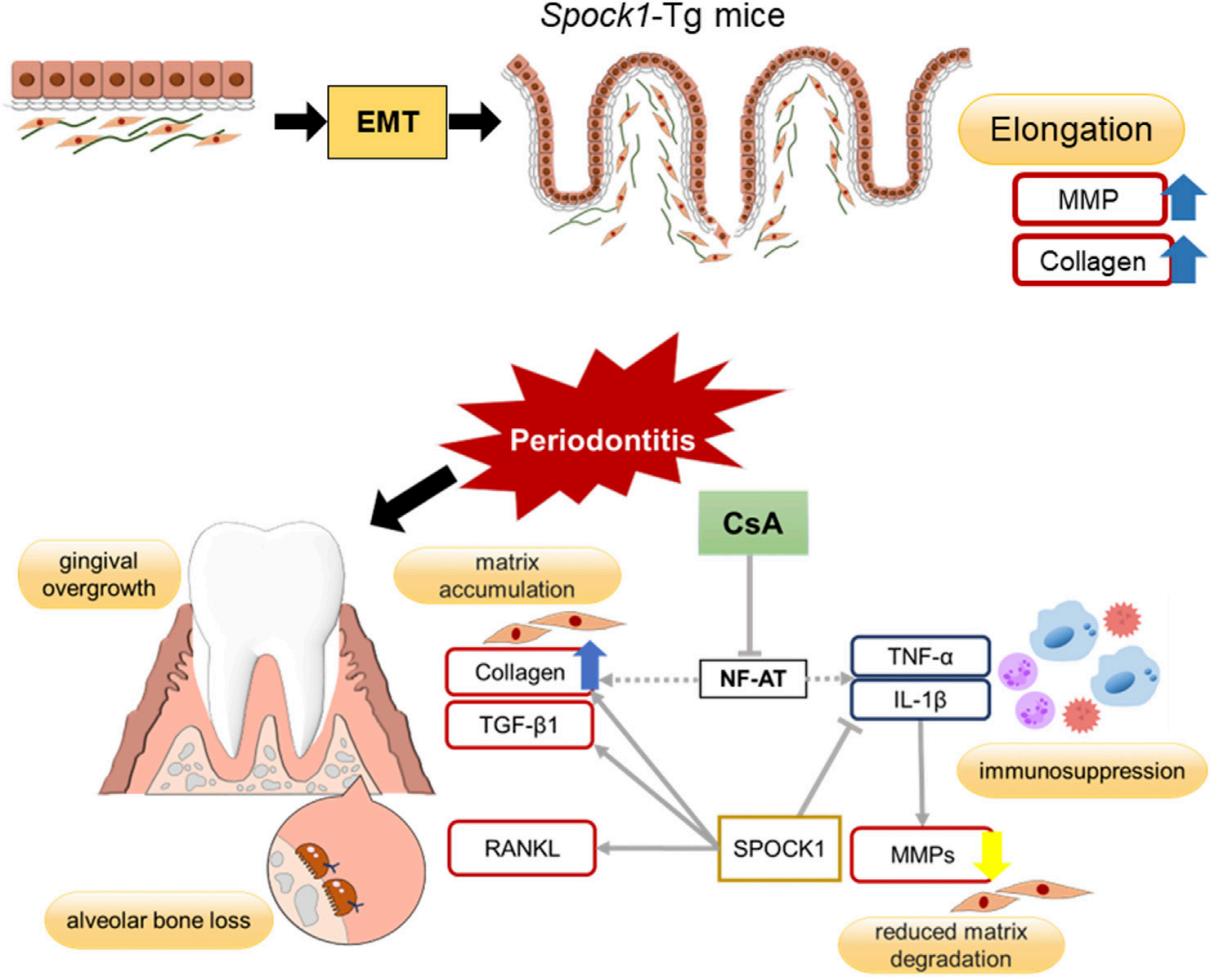
Summary of potential mechanisms of DIGO related to CsA.

In conclusion, EMT, lowered CN/NF-AT activity and inflammatory cytokine expression, which cooperatively contributed to the development of CsA-induced DIGO. It is known that EMT accelerates the elongation of the rete peg into the connective tissue (Okanobu et al., 2017). Inflammation caused by infection usually induces proinflammatory cytokines in immune cells, leading to the production of MMPs in fibroblasts. However, CsA suppresses cytokine production, leading to the suppression of MMPs in fibroblasts. The inhibition of CN/NF-AT signaling causes fibroblasts to produce higher amounts of collagen (Francis and Bai, 2018). All these effects cooperatively contribute to the establishment of the DIGO phenotype. Our hypothetical scheme for explaining the outcome of these networks is demonstrated in Figure 8. The molecular mechanisms by which suppression of CN/NF-AT leads to enhanced collagen production and whether this scenario applies to human DIGO pathology caused by medications other than CsA, such as Ca-channel blockers and phenytoin, need to be elucidated in the near future. Since both chemicals have been reported to suppress calcium ion influx into cells, their ability to suppress CN/NF-AT signaling could be considerable.

## Data Availability

The original contributions presented in the study are included in the article/Supplementary Material, further inquiries can be directed to the corresponding author.
