# Supplementary material for: Epithelial-to-mesenchymal transition, inflammation, subsequent collagen production, and reduced proteinase expression cooperatively contribute to cyclosporin-A-induced gingival overgrowth development
Source: Front Physiol. 2023 Dec 13;14:1298813. doi: 10.3389/fphys.2023.1298813 (PMC10753830; doi:10.3389/fphys.2023.1298813)
Supplement: Supplementary file 1 [file Table1.DOCX]

**Supplemental Table 1**. Antibodies and reagents used in the present study.

| **Product (target)** | **Supplier/Catalog No.** | **Host** | **Dilution** |
| --- | --- | --- | --- |
| Myeloperoxidase | Abcam/ab208670 | Rabbit monoclonal | IF: 1:100 |
| E-cadherin | Santa Cruz/sc8426 | mouse | IF: 1:200 |
| Vimentin | Santa Cruz/sc373717 | mouse | IF: 1:200 |
| MMP2 | Cell Signaling Technology/87809 | Rabbit | IF: 1:1000 |
| MMP9 | Santa Cruz/sc21733 | mouse | IF: 1:200 |
| Anti-rabbit IgG conjugated with Alexa Flour® 488 | Abcam/ab150077 | Goat | IF: 1:200 |
| *E.coli* LPS (O111:B4) | Sigma-Aldrich/L4391 |  | 10 ng/ml |
| Recombinant human RANKL | Peprotech/310-01 |  | 1.0 mg/ml |
| Recombinant human SPOCK1 | R&D Systems/2327-PI-050 |  | 100 μg/ml |

IF: Immunofluorescence

**Supplemental Table 2**. Primers used in this study.

| **RT-qPCR** | **Forward** | **Reverse** |
| --- | --- | --- |
| Mouse 18s rRNA | GCTTAATTTGACTCAACACGGGA | AGCTATCAATCTGTCAATCCTGTC |
| Mouse Spock1 | TGCACGGACAAGGAGCTGCG | GAACCAGTCCTTCAGCCGG |
| Mouse Tnfα | GACAGTGACCTGGACTGTGG | TGAGACAGAGGCAACCTGAC |
| Mouse Il-1β | GAAGAAGAGCCCATCCTCTG | TCATCTCGGAGCCTGTAGTG |
| Mouse Rankl | CGCTCTGTTCCTGTACTTTCG | GAGTCCTGCAAATCTGCGTT |
| Mouse Opg | CCTTGCCCTGACCACTCTTAT | CACACACTCGGTTGTGGGT |
| Mouse Mmp2 | CAGGGAATGAGTACTGGGTCTATT | ACTCCAGTTAAAGGCAGCATCTAC |
| Mouse Mmp9 | AATCTCTTCTAGAGACTGGGAAGGAG | AGCTGATTGACTAAAGTAGCTGGA |
| Mouse Mmp13 | GCCCTGATGTTTCCCATCTA | TTTTGGGATGCTTAGGGTTG |
| Mouse Mmp14 | GTGCCCTATGCCTACATCCG | TTGGGTATCCGTCCATCACT |
| Mouse Tgf-β1 | CCTGTCCAAACTAAGGC | GGTTTTCTCATAGATGGCG |
| Mouse Col1a1 | CATAAAGGGTCATCGTGGCT | TTGAGTCCGTCTTTGCCAG |
| Mouse Col3a | ACAGCTGGTGAACCTGGAAG | ACCAGGAGATCCATCTCGAC |
| Mouse Col4a | GACAGCCAGGTTTGACAGGT | GGCAGCTCTCTCCTTTCTGA |
| Mouse IL-2 | GCGGCATGTTCTGGATTTGACTC | CCACCACAGTTGCTGACTCATC |

All primers were purchased from FASMAC (Kanagawa, Japan).
