# Supplementary figures and images for: Epithelial-to-mesenchymal transition, inflammation, subsequent collagen production, and reduced proteinase expression cooperatively contribute to cyclosporin-A-induced gingival overgrowth development
Source: Front Physiol. 2023 Dec 13;14:1298813. doi: 10.3389/fphys.2023.1298813 (PMC10753830; doi:10.3389/fphys.2023.1298813)

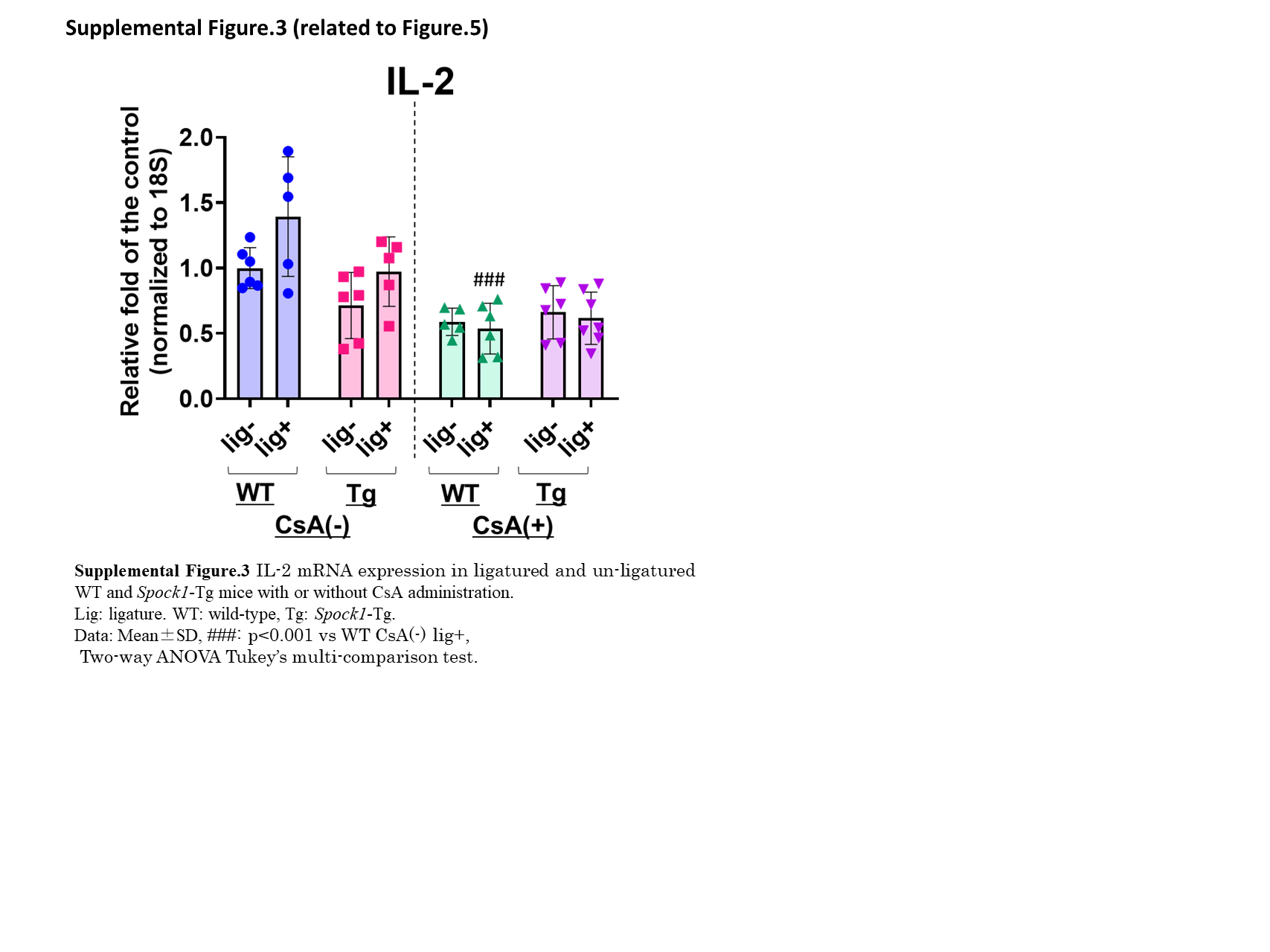

Supplement: Supplementary file 2 [file Image3.TIF]

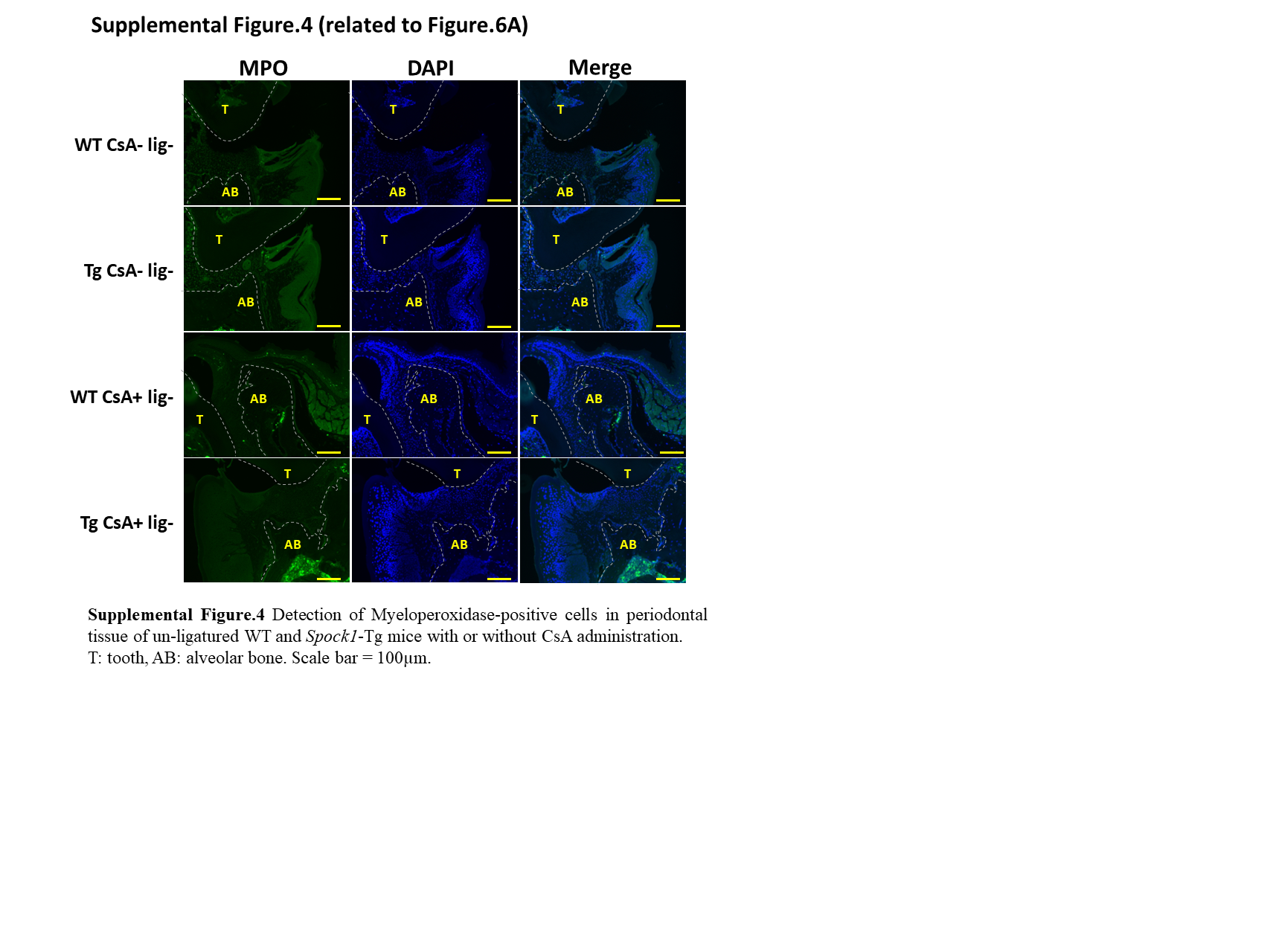

Supplement: Supplementary file 3 [file Image4.TIF]

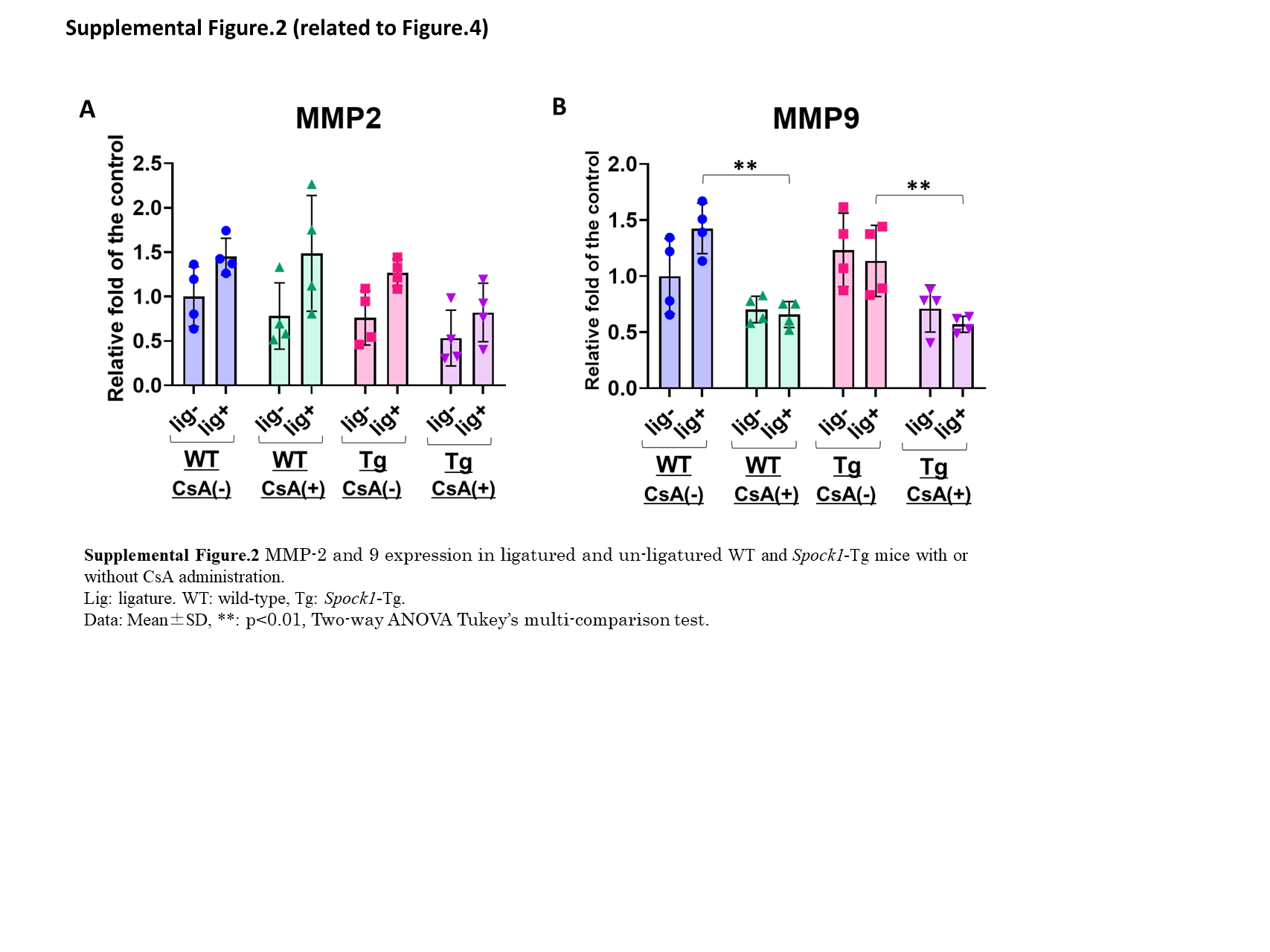

Supplement: Supplementary file 4 [file Image2.TIF]

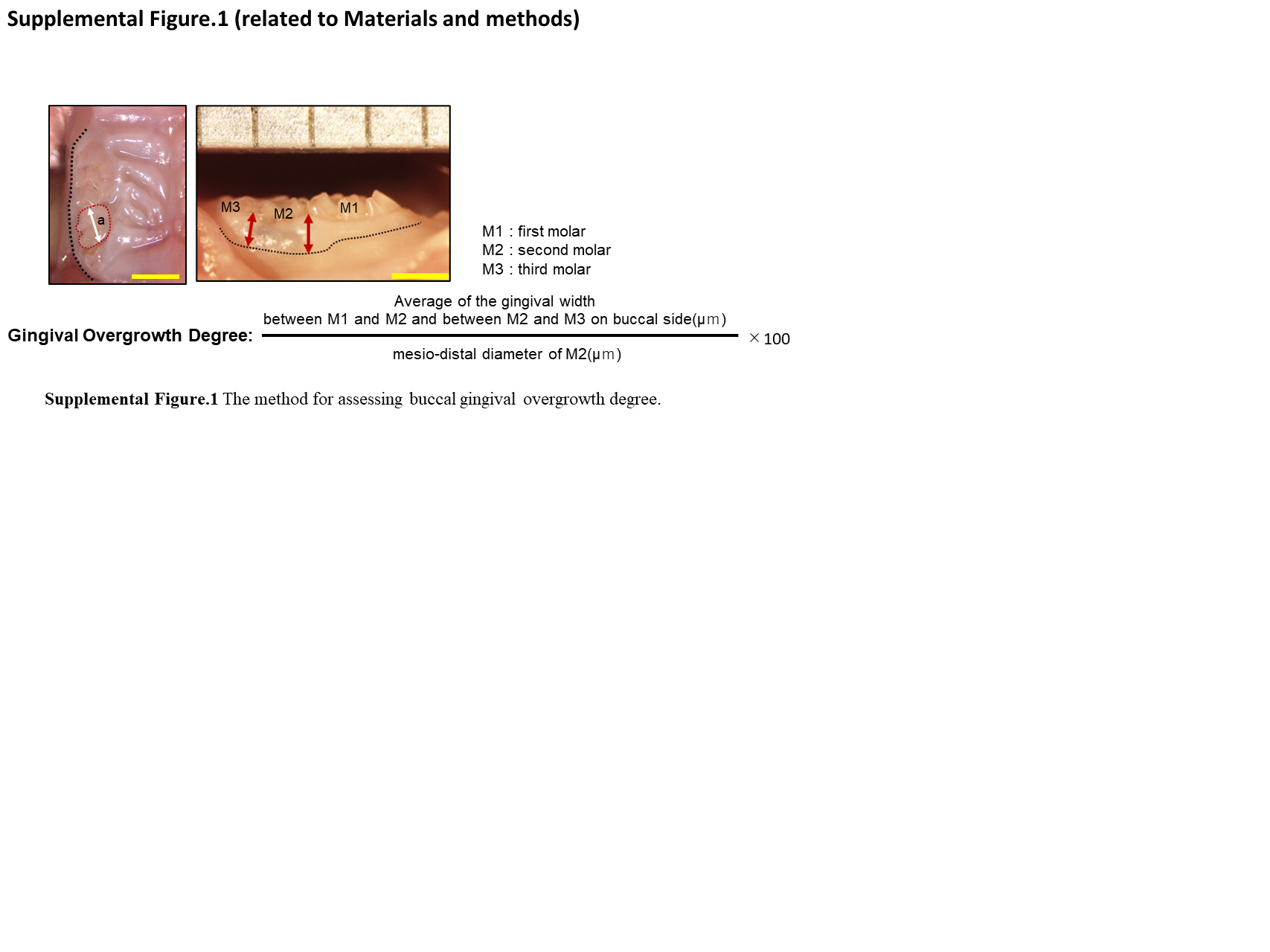

Supplement: Supplementary file 5 [file Image1.TIF]
