# Supplementary material for: Epithelial-to-mesenchymal transition, inflammation, subsequent collagen production, and reduced proteinase expression cooperatively contribute to cyclosporin-A-induced gingival overgrowth development
Source: Front Physiol. 2023 Dec 13;14:1298813. doi: 10.3389/fphys.2023.1298813 (PMC10753830; doi:10.3389/fphys.2023.1298813)
Supplement: Supplementary file 6 [file DataSheet1.docx]

Nutstore sharable link (for revision)

<https://www.jianguoyun.com/c/sd/17fe1a3/6cabe0b34ba46118#from=https%3A%2F%2Fwww.jianguoyun.com%2Fc%2Fsd%2F17fe1a3%2F6cabe0b34ba46118>

A Peer Review Only file

Manuscript ID: 1298813
